# Post-Translational Regulation and Ubiquitination of Brassinosteroid Signaling Components in Plants

**DOI:** 10.3390/cells15201880

**Published:** 2026-10-15

**Authors:** Qingli Jia, Xintong Du, Ting Wang, Zechao Gao, Hui Dong, Ran An, Yuxue Ji, Shihao Wei, Yanfeng Zhang, Shuhua Huang

**Affiliations:** 1 Hybrid Rapeseed Research Center of Shaanxi Province, Yangling 712100, China; jiaqingli@nwsuaf.edu.cn (Q.J.); dh2023@nwafu.edu.cn (H.D.); anrants@nwafu.edu.cn (R.A.); 9202609471@alumni.hainanu.edu.cn (Y.J.); shihao100@126.com (S.W.); zhangyfcl@163.com (Y.Z.); 2 College of Life Sciences, Northwest A&F University, Yangling 712100, China; 2024051221@nwafu.edu.cn (X.D.); zechao_gao@nwafu.edu.cn (Z.G.); 3 College of Agronomy, Northwest A&F University, Yangling 712100, China; tingwang@nwafu.edu.cn

**Keywords:** ubiquitination, BR signaling, E3 ubiquitin ligases, protein stability, regulatory mechanism

## Abstract

Brassinosteroids (BRs), a group of steroidal phytohormones, play vital roles in plant growth and stress responses. The molecular pathways underlying BR signaling have been well characterized. A growing body of data demonstrates that BR signaling components also engage in crosstalk with components from other signaling pathways to modulate plant growth, development, immune defense, and stress adaptation, and such modulation displays BR-dependent or -independent characteristics. Hence, the core components of BR signaling are of paramount importance in the regulation of multiple physiological processes during the entire plant life cycle. As an important post-translational modification, protein ubiquitination is known to precisely modulate the activity and stability of BR signaling components, as well as to govern signal output. This review centers on recent advances and provides a systematic analysis of regulatory factors influencing the stability of core BR signaling components. Particular emphasis is placed on the pivotal role of E3 ubiquitin ligases in governing the degradation, accumulation, and functional activity of these components via ubiquitination, while also contrasting the functional conservation and divergence of relevant E3s between Arabidopsis and other crops. Further dissection of the molecular regulation of BR signaling core components will facilitate a better understanding of the underlying signaling network and enable the development of strategies for crop growth regulation, yield promotion, and stress adaptation.

## 1. Introduction

**Figure 1:**
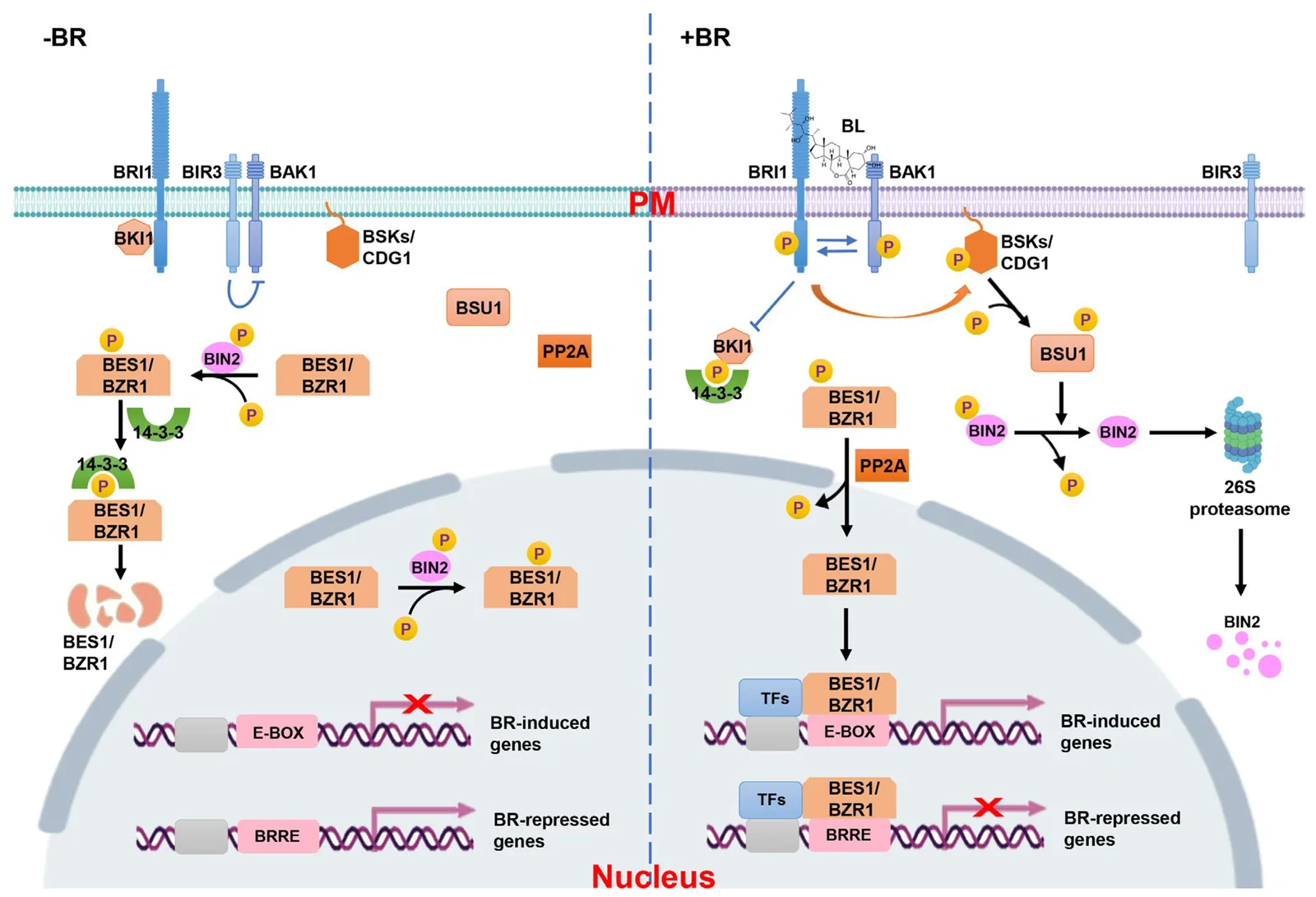
The model of the brassinosteroid signaling pathway in *Arabidopsis*. Under BR-deficient conditions (**left**) panel, the activities of BRI1 and BAK1 are suppressed by multiple regulatory proteins, among which BKI1 and BIR3 are included. Such processes disrupt the physical association of BRI1 with BAK1 and sustain the constitutively kinase-active, phosphorylated form of BIN2. Active BIN2 further phosphorylates transcription factors BES1 and BZR1 to suppress their DNA-binding competence. 14-3-3-protein binding sequesters phosphorylated BES1 and BZR1 in the cytosol and facilitates their subsequent breakdown. In the presence of brassinosteroids, exemplified by the brassinolide (**right**) panel, ligands associate with the extracellular domains of BRI1 and BAK1 and trigger early BR signaling. Such binding promotes the detachment of inhibitory regulators BKI1 and BIR3 and enables sequential phosphorylation within the BRI1-BAK1 complex. Upon full activation, BRI1 directly phosphorylates BSKs and CDG1 to activate downstream BSU1. Active BSU1 removes phosphate groups from BIN2 and accelerates its degradation by the 26S proteasome. In the meantime, phosphorylated BKI1 interacts with 14-3-3 proteins and releases BES1 and BZR1. PP2A-mediated dephosphorylation triggers their nuclear translocation, allowing these transcription factors to bind target-gene promoters and govern BR-responsive transcripts.

In nature, unlike animals, plants lack nervous systems and the ability to move actively. Consequently, they have evolved diverse signaling molecules to perceive environmental cues, coordinate growth, adapt to various stresses, and complete their life cycles [1]. Among these molecules are plant hormones, a class of naturally occurring trace organic signaling compounds [2]. Although they do not directly participate in cellular structural construction or energy metabolism, plant hormones serve as core regulators of plant physiological activities. Brassinosteroids (BRs), a group of polyhydroxylated sterol-derived plant hormones, play pivotal roles in modulating plant growth, development, and stress responses. As essential phytohormones widely distributed across plant tissues, BRs govern multiple cellular events including cell division, cell elongation and cell differentiation, and also participate in programmed cell death. They orchestrate the whole-plant life cycle ranging from seed germination, vegetative growth, floral transition and fruit formation, through to organ senescence and abscission, thus sustaining orderly plant morphogenesis [3,4,5]. As a central hub for environmental signal transduction, BR signaling mediates adaptive responses to external stimuli including light, temperature, water status, salt stress, and pathogen and pest attacks [4,5,6]. It modulates stomatal movement, photosynthate allocation, osmolyte biosynthesis, and the expression of stress-responsive genes, thus improving plant stress resistance and survival potential [4,5]. Nowadays, the BR signaling transduction pathway has been well characterized in *Arabidopsis thaliana* (*Arabidopsis*), *Oryza sativa* (rice), and numerous other crops (Figure 1).

### 1.1. BR Signaling in Plants

Plants perceive the BR signals via a cell membrane-localized receptor, BRASSINOSTEROID INSENSITIVE 1 (BRI1) [7,8,9,10]. In the absence of BRs, BRI1 remains inactive due to the auto-inhibitory function of its C-terminal domain, autophosphorylation in the kinase domain [11], and the associations with BRI1 KINASE INHIBITOR1 (BKI1) [12,13,14], *BOTRYTIS*-INDUCED KINASE 1 (BIK1) [15], and SUPPRESOR of BRI1 (SBI1) [16]. Meanwhile, the complex formed by BRI1 with its co-receptors, SOMATIC EMBRYOGENESIS RECEPTOR KINASEs (SERKs) [17,18,19] such as BRI1-ASSOCIATED RECEPTOR KINASE 1 (BAK1), is inhibited by BAK1-INTERACTING RECEPTOR-LIKE KINASE 3 (BIR3) and MEMBRANE STEROID-BINDING PROTEIN 1 (MSBP1) in *Arabidopsis*, by OsREM4.1 in rice, and by DROOPY LEAF1 (DPY1) in *Setaria italica* (foxtail millet), thereby suppressing the initiation of early BR signaling [20,21,22,23]. The glycogen synthase kinase 3 (GSK3)-like kinases, including BRASSINOSTEROID INSENSITIVE2 (BIN2), are core negative regulators of the BR signaling pathway [24,25]. They phosphorylate the transcription factors BRASSINAZOLE RESISTANT 1 (BZR1)/BRI1 EMS SUPPRESSOR 1 (BES1), promoting their interaction with 14-3-3 proteins, which in turn leads to their cytoplasmic retention and inhibits the activation of downstream BR-responsive genes [26,27,28,29].

In the presence of BRs, BRI1 recognizes and binds BL, the most active form of BRs, through its extracellular domain. BRI1 then associates with BAK1 to form a BRI1–BL–BAK1 complex, which further promotes the assembly of an intracellular complex between BRI1 and BAK1, resulting in the release of negative regulators from BRI1 and the full activation of BRI1 via sequential phosphorylation of BAK1 [8,9,10,30,31,32]. Fully activated BRI1 associates with BR SIGNALING KINASES (BSKs) and CONSTITUTIVE DIFFERENTIAL GROWTH 1 (CDG1) and phosphorylates them to trigger their activation [25,33]. The activated BSKs and CDG1 then phosphorylate and activate BRI1-SUPPRESSOR 1 (BSU1) [25,33]. BSU1 interacts with BIN2 to suppress its kinase activity and BIN2-dependent phosphorylation of BES1/BZR1 [25,34]. Subsequently, BR-activated PROTEIN PHOSPHATASE 2A (PP2A) dephosphorylates BES1/BZR1 and inhibits their interaction with 14-3-3 proteins [26,27,35], thereby promoting the nuclear accumulation of BES1/BZR1 facilitated by IMPORTIN-β4 (IMB4) [36]. BES1/BZR1 then recruits BES1-INTERACTING MYC-LIKE 1 (BIM1) [37], TOPLESS (TPL) [38], BRAHMA-associated SWI/SNF complexes (BAS) [39], and several other proteins to coordinately regulate the expression of BR-responsive genes [40,41,42]. In addition to the negative regulatory role of BKI1 in the BR signaling pathway, BKI1 also exerts a positive regulatory function within the BR signaling cascade [43]. In the absence of BRs, BKI1 and 14-3-3 proteins inhibit the BR receptor BRI1 and the transcription factor BES1/BZR1, respectively. BR signaling promotes the phosphorylation of BKI1; phosphorylated BKI1 interacts with 14-3-3 proteins and dissociates into the cytoplasm, thereby relieving the inhibition of BRI1 and BES1/BZR1 and facilitating downstream BR signal outputs [43].

When BR signaling is impaired, BR-insensitive mutants exhibit phenotypes similar to those of BR biosynthesis-deficient mutants, including small and rounded leaves, dwarf plant stature, and male sterility [25]. By contrast, constitutive activation of BR signaling exerts numerous adverse effects on plant growth and development. For instance, Overexpression of *BRI1* in a transgenic background already overexpressing *PMEI5* led to enhanced BR signaling and gave rise to severely dwarfed plants with twisted organ morphology [44,45,46]. This positive effect on BR signaling was also observed upon overexpression of *SlBRI1* in *Solanum lycopersicum* (tomato), yet this came at the cost of reduced drought tolerance [47]. An overdose of *BAK1* in *Arabidopsis* causes leaf necrosis and growth defects accompanied by increased BR signaling output [48,49], which is distinct from the cell death phenotype observed in the *bak1 bkk1* double null mutant, lacking both *BAK1* and its closest homolog *BAK1-LIKE 1* (*BKK1*) [50,51,52,53,54]. At the other end of the functional spectrum, the BR-hypersensitive mutant *elg-D* (an allele of *BAK1*) displays constitutive BR signaling activation, coupled with enhanced vulnerability to bacterial pathogen infection and salt stress [55,56]. The *bin2-3 bil1 bil2* triple mutant resulting from the simultaneous knockout of *BIN2*, *BIN2-LIKE1* (*BIL1*), and *BIN2-LIKE2* (*BIL2*) shows BR signaling hyperactivation, extreme dwarfism, and organ distortion [57]. Notably, recent research indicates that this mutant exhibits impaired female gametophyte development [58], compromised drought tolerance [59], and decreased resistance to *Pseudomonas syringae* [60]. The dominant mutant *bes1-D* (*BRI1-EMS-SUPPRESSOR 1*) exhibits constitutively activated BR signaling, leading to increased petiole elongation and curly leaves [28], whereas subsequent studies have shown that it displays reduced tolerance to both drought and salt stress [61,62,63]. A dominant mutation in *BZR1* (present in *bzr1-1D*), a close homolog of *BES1*, promotes the nuclear accumulation of dephosphorylated BZR1 protein and triggers its constitutive activation, thereby resulting in constitutive BR responses [29]. Although *bzr1-1D* is constitutively active, it paradoxically activates a feedback inhibitory pathway, leading to reduced endogenous BR levels and a semi-dwarf plant architecture [29]. These observations indicate that either insufficient or excessive BR signaling output can disrupt normal plant development and stress responses. Therefore, the components of the BR signaling pathway are subject to both positive and negative regulation in plants to maintain BR signaling intensity at an appropriate level, thereby ensuring normal plant growth and development.

Post-translational modifications, such as dephosphorylation, phosphorylation, and ubiquitination, play important roles in either activating or attenuating plant hormone and pattern-triggered immunity (PTI) signaling intensity [4,64,65,66,67,68,69,70,71]. Among these, the role of dephosphorylation and phosphorylation in modulating the BR signaling pathway has been particularly well documented [4]. Accumulating recent evidence indicates that endocytosis and protein degradation are also critical for controlling BR signaling output and the stability of BR components, with ubiquitination serving as a key regulatory mechanism in these processes.

### 1.2. The Ubiquitin–Proteasome System

**Figure 2:**
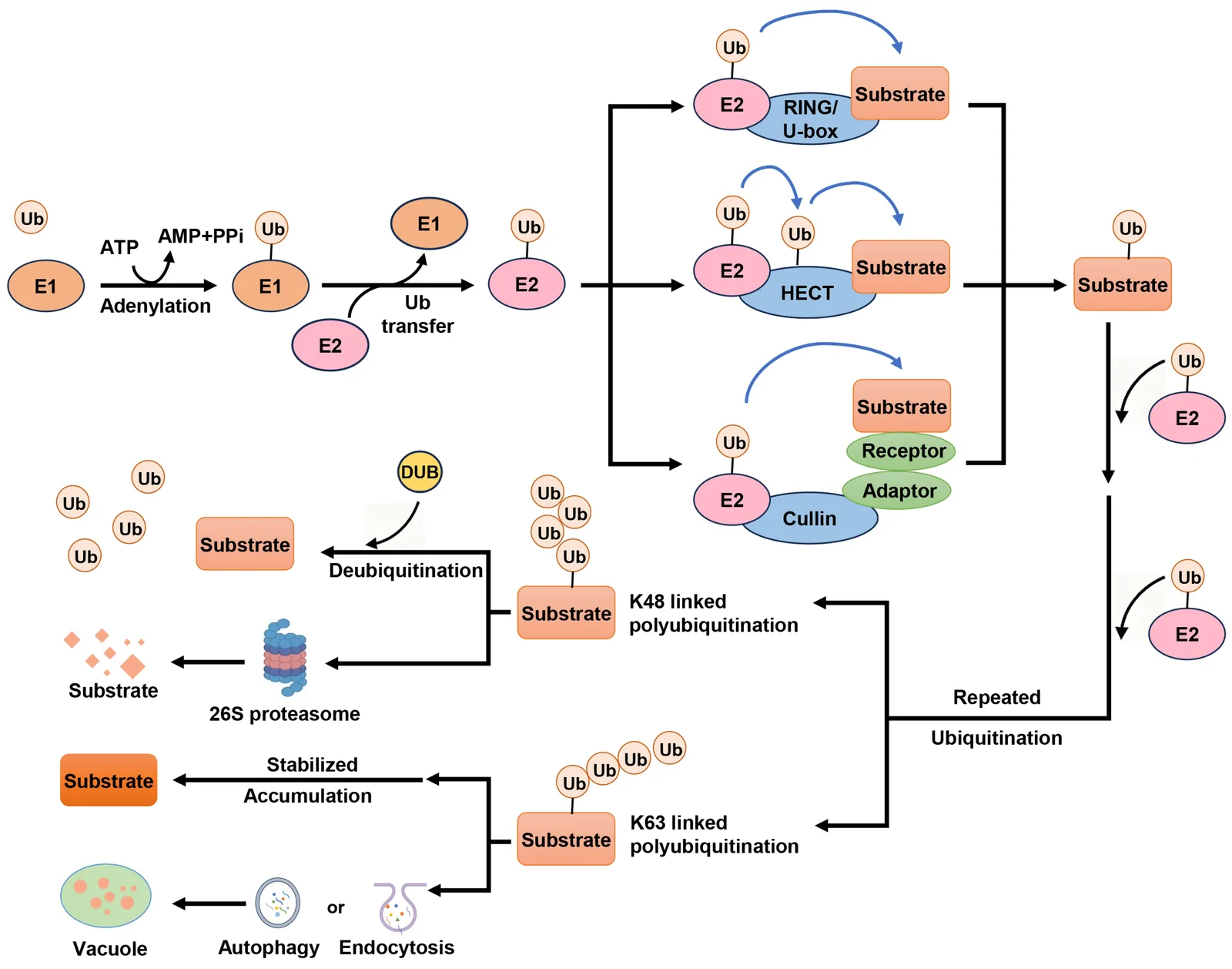
The ubiquitin-dependent protein-degradation system. The E1 ubiquitin-activating enzyme conjugates with ubiquitin to generate the E1 Ub complex in an ATP-dependent reaction. Subsequent to E1-mediated activation, ubiquitin is relayed to the E2 ubiquitin-conjugating enzyme and forms the E2 Ub complex. E2 and E3 enzymes function collaboratively to transfer ubiquitin to E3-recruited substrates. RING/U box type and Cullin-dependent E3s mediate direct ubiquitin delivery, whereas HECT-type E3s operate via an indirect pathway. After multiple cycles, E2 and E3 enzymes promote further ubiquitin attachment to the substrate and consequently assemble polyubiquitin chains. Protein fate is governed by the linkage type of assembled polyubiquitin chains. K48-linked polyubiquitinated proteins are generally targeted for degradation by the 26S proteasome. Alternatively, deubiquitinating enzymes (DUBs) can remove polyubiquitin chains to recycle these substrates. K63-polyubiquitinated substrates can undergo autophagic- and endocytosis-driven vacuolar breakdown, yet some proteins modified with K63-linked polyubiquitin chains show heightened stability and intracellular accumulation in vivo.

Ubiquitin is a highly conserved small polypeptide in eukaryotes consisting of 76 amino acid residues. It modifies lysine residues of target proteins through covalent conjugation, a process termed ubiquitination [72]. Ubiquitination is sequentially catalyzed by three types of enzymes: ubiquitin-activating enzymes (E1s), ubiquitin-conjugating enzymes (E2s), and ubiquitin ligases (E3s). First, E1 activates ubiquitin in an ATP-dependent manner. Subsequently, the activated ubiquitin is transferred to E2. Finally, E3 ubiquitin ligases recognize specific substrates and facilitate the attachment of ubiquitin from E2 to target proteins (Figure 2) [73]. The *Arabidopsis* genome encodes 2 E1, at least 37 E2, and over 1400 E3 ubiquitin ligases [73]. The abundance of E3 proteins underscores the critical function of ubiquitination in modulating plant growth, development, and stress responses. Based on their conserved catalytic domain architectures, ubiquitin transfer mechanisms, and subunit composition, E3 ubiquitin ligases are mainly classified into really interesting new gene (RING), homologous to E6-AP C-terminus (HECT), U-box, and Cullin-RING (CRL) types [66]. Among them, CRLs function as multi-subunit complexes, whose core components consist of a Cullin scaffold protein, an RBX1 protein, and a variable module responsible for substrate specificity; additional auxiliary subunits may also be included [73]. According to distinct cullin scaffold proteins and substrate recognition mechanisms, CRLs are divided into four major subtypes: SCF, BTB, DDB, and APC E3 ligases [71,73]. These subtypes recognize their respective substrates by means of F-box, BTB, DWD, and several interchangeable recognition subunits [73].

The functional consequences of ubiquitination differ depending on the ubiquitination mode (monoubiquitination, multiubiquitination, and polyubiquitination), ubiquitin chain linkage type (e.g., K48, K63), and ubiquitin chain length [72,73,74]. Monoubiquitination and multiubiquitination primarily function to modify protein activity and regulate subcellular protein localization, whereas polyubiquitination usually mediates the selective degradation of target proteins through the ubiquitin–26S proteasome system [74,75]. The ubiquitin molecule harbors seven lysine residues (K6, K11, K27, K29, K33, K48, and K63), and its N-terminal methionine (M1) can also participate in chain assembly, enabling eight distinct polyubiquitin chain linkages. Among these, K48- and K63-linked chains are the most extensively characterized. K48-linked chains serve as the canonical signal for proteasomal degradation, targeting proteins for elimination by the 26S proteasome [72,74,75]. This pathway efficiently eliminates damaged, misfolded, or physiologically obsolete proteins, playing a central role in fundamental biological processes such as cell cycle progression, protein quality control, and immune surveillance. In plants, K48-dependent protein degradation is widely involved in multiple pathways, including growth and development regulation, hormone signaling transduction, and stress response [73,74]. In contrast, K63-linked ubiquitination primarily executes non-degradative signaling regulatory functions and is extensively involved in various biological processes, including maintaining protein homeostasis, mediating endocytic trafficking, and activating MAPK signaling cascades upon pathogen infection [75]. Furthermore, K63 ubiquitination also regulates the interactions of multiple protein complexes during DNA repair and cell cycle progression, which is of great significance for plant cell survival and homeostasis maintenance under stress conditions [75,76]. It is generally believed that E2 enzymes are the key determinants of ubiquitin chain linkage types, and E2 enzymes act in synergy with E3 enzymes to jointly confer specificity to the ubiquitination reaction (Figure 2) [77,78,79,80,81].

Recent years have seen extensive reports on the ubiquitination of BR receptors and BR signaling pathway components (Table 1). Ubiquitination not only plays a key role in regulating BR signaling but also participates in multiple other signaling pathways mediated by BR signaling components. Focusing on *Arabidopsis*, rice, and other major crops, this review systematically discusses the regulatory roles of ubiquitination and protein stability throughout the entire BR signaling cascade, from ligand perception by BRI1 to downstream physiological responses controlled by BKI1, BAK1, BIK1, BSKs, BIN2, and BES1/BZR1 (Figure 1), and also addresses the involvement of BR signaling components in other signaling pathways.

## 2. Ubiquitination in BRI1 Signaling

**Figure 3:**
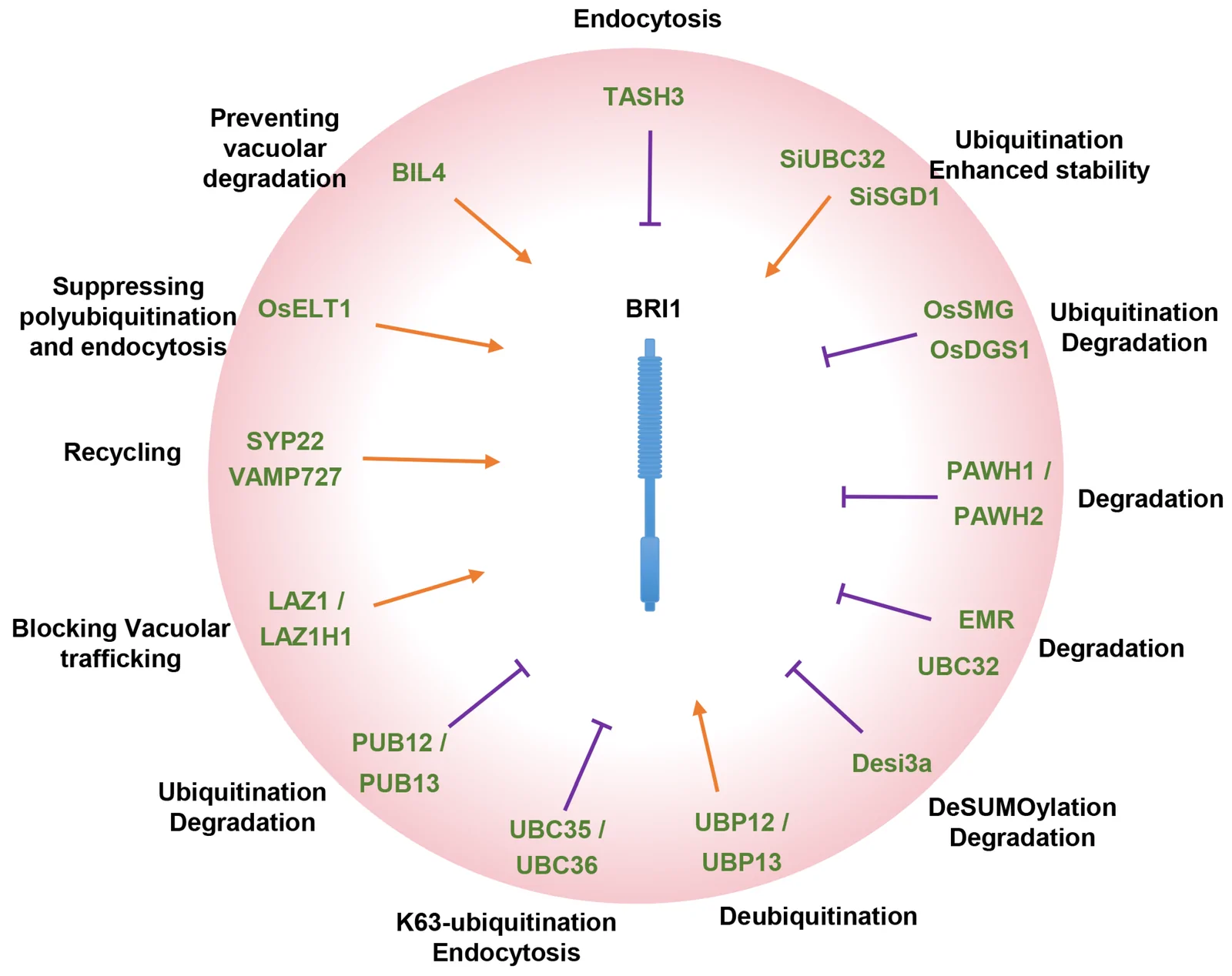
Regulation of BRI1 stability by ubiquitin-associated regulators. TASH3 interacts with ubiquitinated BRI1 and promotes its endocytosis. BIL4 and OsETL1 promote BR signaling by suppressing vacuolar localization and degradation of BRI1. SYP2 and VAMP727 interact with BRI1 and mediate recycling of internalized BRI1 back to the PM, thereby boosting BR signaling. LAZ1 and LAZ1H1 repress vacuolar trafficking and degradation of BRI1. PUB12/PUB13 and UBC35/UBC36 ubiquitinate BRI1 to trigger its endocytosis and degradation, whereas UBP12/UBP13 deubiquitinate BRI1. Desi3a deSUMOylates BRI1 and promotes its ubiquitination and degradation. EMR and UBC32 attenuate BR signaling via degradation of misfolded BRI1; a similar mechanism is observed for OsSMG3 and OsDGS1 in rice, as well as for PAWH1 and PAWH2 in Arabidopsis. In contrast, SiUBC32 and SiSGD1 promote BR signaling by ubiquitinating BRI1 and enhancing its stability. The T-shaped lines represent negative regulation of target protein stability and accumulation, whereas arrows represent positive regulation of target protein stability and accumulation.

*Arabidopsis* BRI1, the leucine-rich repeat receptor-like kinase (LRR-RLK), detects BRs [7]. The most active form of BRs, BL, functions as the primary signaling molecule that initiates the BR signaling cascade upon binding to BRI1, which then forms a heterodimer with BAK1 and is fully activated through transphosphorylation to initiate downstream signaling [31]. In addition to phosphorylation, ubiquitination represents a critical post-translational modification that regulates the stability of the BR receptor BRI1 and modulates BR signaling output (Figure 3 and Table 1).

It was verified that, in *Arabidopsis*, BRI1 undergoes K63-linked polyubiquitination in vivo in a manner largely independent of BR ligand binding but dependent on BRI1 kinase activity and the co-receptor BAK1 [140]. The ubiquitination promotes BRI1 endocytosis from the plasma membrane and serves as a critical signal for its recognition by the trans-Golgi network/early endosomes (TGN/EE), which mediates vacuolar targeting and sorting. This endocytosis process of the ubiquitinated BRI1 probably depends on the plant-specific TPLATE adaptor complex, including the TSAUCER (TASH3) subunit [82,141,142]. Through this mechanism, K63-linked polyubiquitination negatively regulates BRI1-mediated BR signaling [140]. Ubiquitination deficiency causes BRI1 retention at the plasma membrane, impaired endocytosis and vacuolar sorting, and consequently enhanced BR signaling output [140]. Negative regulators of BRI1 endocytosis and vacuolar sorting have also been reported. For example, in *Arabidopsis*, the BRASSINAZOLE (BRZ)-INSENSITIVE-LONG HYPOCOTYL4 (BIL4) localizes to the TGN/EE, where it directly interacts with BRI1 in endosomes and stabilizes BRI1 by preventing its vacuolar degradation, thus promoting BR signaling [83]. In rice, a kinase-dead RLK, ENHANCED LEAF INCLINATION AND TILLER NUMBER 1 (ELT1), localizes to the plasma membrane and specifically binds the intracellular domain of BRI1 [84]. By inhibiting the potential K63-linked polyubiquitination of BRI1, ELT1 reduces BRI1 endocytosis and vacuolar degradation, resulting in BRI1 accumulation at the plasma membrane [84]. Additionally, the SNARE-complex components SYNTAXIN OF PLANTS 22 (SYP22) and VESICLE-ASSOCIATED MEMBRANE PROTEIN 27 (VAMP727) directly interact with BRI1 and specifically mediate the recycling of internalized BRI1 back to the plasma membrane, thus maintaining the plasma membrane receptor pool [85,143]. Meanwhile, the tonoplast-localized DUF300 domain-containing proteins LAZARUS1 (LAZ1) and LAZ1 HOMOLOG143 (LAZ1H1) maintain vacuolar integrity to indirectly regulate BRI1 homeostasis. Loss of both genes results in aberrant vacuolar morphology and excessive BAK1 accumulation at the tonoplast, which accelerates BRI1 vacuolar trafficking and degradation [86].

Currently, E3 ubiquitin ligases that participate in the ubiquitination and regulation of BRI1 have also been identified in plants. Following BR perception, BRI1 is activated [31] and phosphorylates plant U-box (PUB) E3 ligases PUB12 and PUB13 at Ser344, which enhances the interaction between PUB12/PUB13 and BRI1 and promotes BRI1 ubiquitination, endocytosis, and degradation [87]. In the *pub12 pub13* double mutant, BRI1 ubiquitination is reduced, endocytosis is impaired, and BRI1 retention at the plasma membrane is prolonged, ultimately leading to BR hypersensitivity [87]. This phenotype resembles that of the ubiquitination-deficient BRI1 mutant, BRI1_25KR_ [87,140]. Current research has identified UBIQUITIN CONJUGATING ENZYME 35 (UBC35) and UBC36 as the core E2 enzymes responsible for generating K63-linked ubiquitin chains in *Arabidopsis* and mediating the K63 ubiquitination of BRI1 [75,144]. This modification serves as an essential signal for the initiation of BRI1 endocytosis and its subsequent vacuolar degradation via vesicular trafficking [75,76]. In the *ubc35 ubc36* double mutant, reduced ubiquitination of BRI1 impairs endocytosis and leads to protein accumulation, resulting in hyperactivation of BR signaling and disrupting developmental and hormonal homeostasis [75]. However, the specific E3 ligase responsible for cooperative K63-linked BRI1 ubiquitination via interaction with UBC35 and UBC36 has not yet been characterized experimentally. Notably, the ubiquitination of BRI1 is governed by two deubiquitinating enzymes, UBP12 and UBP13, which directly bind to BRI1 and remove its K63-linked polyubiquitin chains [88]. This inhibits the vacuolar trafficking and degradation of BRI1, thereby maintaining BRI1 protein stability and positively regulating BR signaling [88]. In addition to ubiquitination, BRI1 can be SUMOylated at residues K1066 and K1118 under low-temperature conditions. This modification competes with ubiquitination for the same residues and inhibits BRI1 degradation. High temperature triggers DESUMOYLATING ISOPEPTIDASE 3a (Desi3a)-mediated deSUMOylation of BRI1, which promotes ubiquitination-dependent degradation of BRI1, thereby attenuating BR signaling [89].

Newly synthesized BRI1 is folded and glycosylated in the endoplasmic reticulum, while misfolded BR receptors, including *bri1-5*, *bri1-9,* and *bri1-301*, are retained in the endoplasmic reticulum (ER) and ubiquitinated through the ER-associated degradation (ERAD) pathway and subsequently eliminated by proteasome-mediated degradation [91,145,146]. ERAD-mediating RING finger protein (EMR), a plant-specific RING-type E3 ubiquitin ligase, is localized to the ER and interacts with the E2 enzyme UBIQUITIN CONJUGATING ENZYME 32 (UBC32) [90]. As a core component of the ERAD system, EMR mediates the degradation of the misfolded protein bri1-5. Mutation of EMR leads to accumulation of the misfolded bri1-5 protein and partially rescues the BR signaling defect of the *bri1-5* mutant [90]. Protein Associated With Hrd1-1 (PAWH1) and PAWH2 are plant-specific components of the endoplasmic reticulum-associated degradation (ERAD) pathway [91]. Both proteins localize to the ER membrane and indirectly bind the E3 ubiquitin ligase HMG-CoA reductase degradation 1 (Hrd1) via EMS-MUTAGENIZED BRI1 SUPPRESSOR 7 (EBS7) [91]. In the *pawh1 pawh2* double mutant, the stability of EBS7 and Hrd1 proteins is significantly reduced, leading to impaired degradation of ERAD substrates such as bri1-5 and bri1-9 [91]. A similar ERAD mechanism has also been documented in rice. The SMALL GRAIN 3 (SMG3) protein, a homolog of the *Arabidopsis* E2 enzyme UBC32, was shown to interact with DECREASED GRAIN SIZE 1 (DGS1), a C3HC4-type RING E3 ligase, within the endoplasmic reticulum [90,92]. This complex was found to ubiquitinate misfolded or incompletely folded forms of OsBRI1, such as bri1-101, leading to their elimination via the proteasome [147]. Consequently, this complex positively regulates BR signaling and promotes grain growth [92,147]. In contrast, in foxtail millet, the RING-type E3 ligase SMALL GRAIN AND DWARF1 (SGD1) and its cognate E2 SiUBC32 form a conserved E2-E3 functional module mainly on the plasma membrane and ER. This regulatory module is highly conserved among cereal crops, including rice, maize, and wheat [93]. SGD1 directly binds and ubiquitinates the BR receptor SiBRI1, markedly enhancing SiBRI1 stability and BR signaling output, thereby controlling grain size and yield through the regulation of glume cell expansion [93]. Phylogenetic analysis shows that SGD1 is the ortholog of rice THERMO-TOLERANCE 3.1 (TT3.1), a protein known to positively regulate thermotolerance in rice [148]. Furthermore, three homologs of SGD1 were identified in *Arabidopsis*, with PPRT1 participating in salt stress response [149]. Building on these observations, the SGD1-BRI1 module shows considerable promise for improving crop yield under stress conditions. This notion is corroborated by evidence from tomato, where SlBRI1 has been implicated in responses to both heat and salt stress [150,151].

Recent progress has advanced our understanding of BRI1 ubiquitination in BR signaling, yet key gaps remain, including its exact ubiquitination sites, ubiquitin-chain types, and the molecular switch between degradative and non-degradative ubiquitination. Crosstalk between ubiquitination and other post-translational modifications such as phosphorylation and SUMOylation also needs further exploration. Manipulating BRI1 ubiquitination offers a promising strategy for crop molecular breeding, and future studies combining multi-omics and gene-editing tools will help resolve these open questions and promote translational applications.

## 3. Ubiquitination in BKI1 Signaling

BKI1, an intrinsically disordered protein, was initially identified as a negative regulator of BR signaling [13,14,152]. An *Arabidopsis* U-box E3 ubiquitin ligase, PUB30, has been shown to interact with BKI1, leading to its ubiquitination and degradation [94]. In the *pub30* mutant, BKI1 degradation is slowed, resulting in increased BKI1 accumulation, attenuated BR signaling, and enhanced salt tolerance [94]. In wheat, *ZnF* encodes a plasma membrane-localized RING-type E3 ubiquitin ligase that directly interacts with both TaBKI1 [95] and TaBRI1 [95,153,154]. In the absence of BR, ZnF preferentially binds TaBRI1, keeping the ZnF–TaBKI1 interaction weak and TaBKI1 stable to inhibit TaBRI1. Upon BR perception, TaBRI1 undergoes conformational changes, weakening ZnF–TaBRI1 binding while strengthening ZnF–TaBKI1 interaction [95]. This switch allows ZnF to ubiquitinate and degrade TaBKI1, releasing TaBRI1 from inhibition. ZnF does not directly target TaBRI1 for ubiquitination; instead, it fine-tunes BR signaling by controlling TaBKI1 ubiquitination and stability, thereby regulating wheat plant height, nitrogen-use efficiency, and grain yield [95]. This study establishes a novel breeding strategy for semi-dwarf and high-yield wheat based on the regulation of BR signaling (Table 1).

BKI1 is not exclusively involved in the BR pathway but serves as a core hub protein that simultaneously regulates multiple receptor kinase signaling pathways and responds to various nutrient signals. BKI1 also simultaneously inhibits the activity of ERECTA receptor kinase, thereby controlling plant architecture and peduncle orientation through coordinated regulation of the BR and ER pathways [155]. Activation of BR signaling promotes the dissociation of BKI1 from the plasma membrane, thereby simultaneously relieving the inhibition of both BRI1 and ER, leading to crosstalk between the two pathways [155]. In roots, BKI1 responds to low nitrogen (N), phosphorus (P), and iron (Fe) nutritional cues and mediates auxin–BR crosstalk: low nitrogen and low iron conditions suppress BKI1 expression to activate BR signaling, promoting primary root elongation [156,157]. Conversely, low phosphorus induces iron accumulation, which upregulates BKI1 and inhibits BR signaling, leading to root growth arrest [157]. By integrating hormonal and nutritional signals at the levels of translation and membrane localization, BKI1 serves as a central hub governing plant architecture establishment and root plasticity. The PvRXLR131 effector protein secreted by *Plasmopara viticola* directly and specifically binds to host BKI1 (VvBKI1, AtBKI1, NbBKI1) on the plasma membrane [158]. This stabilizes plasma membrane-localized BKI1 and suppresses both the BR and ER signaling pathways, thereby inhibiting plant PTI, elicitor-induced cell death, and disease resistance [158]. Silencing *BKI1* completely abolishes the virulence function of this effector [158]. By interacting with BKI1 and anchoring it on the plasma membrane, the tomato leaf curl Yunnan virus (TLCYnV) C4 protein blocks BKI1/ER complex dissociation, thereby compromising ER autophosphorylation and dampening the downstream MAPK cascade. This interference ultimately fosters a host environment that favors viral infection [159].

Given the diverse and pleiotropic functions of BKI1, the E3 ubiquitin ligases that control BKI1 protein stability are largely unidentified in plants. It also remains unclear whether these E3s mediate degradative or non-degradative ubiquitination of BKI1, and how their activities are coordinated with BR signaling and other developmental or environmental cues. Further identification and functional characterization of such E3 ligases will deepen our understanding of the multilayered regulatory network governing BKI1 and BR signal transduction.

## 4. Ubiquitination in BAK1 Signaling

BAK1, also known as SOMATIC EMBRYOGENESIS RECEPTOR KINASE3 (SERK3), is a highly conserved LRR-RLK in *Arabidopsis* that functions as a core co-receptor integrating two major pathways: plant growth and development [4,160] and innate immunity [161,162,163,164]. Furthermore, BAK1 is involved in regulating cell death homeostasis; its deficiency leads to autoimmunity and programmed cell death [23,51,165,166,167,168]. By dynamically assembling distinct receptor complexes and precisely transmitting phosphorylation signals, BAK1 serves as a key molecular switch that balances plant growth and defense [163].

The ubiquitin/proteasome system is a ubiquitous protein degradation pathway in cells. Upon specific inhibition of proteasome activity by MG132, BAK1 protein accumulation did not change significantly, indicating that under the experimental conditions used, BAK1 is not degraded via the ubiquitin/26S proteasome pathway [100]. The protein stability of BAK1 is co-regulated by plant developmental signals and immune signals [23,48,169]. BAK1 undergoes Ca^2+^-dependent proteolytic cleavage in plants, a process that can be induced by pathogen/microbe-associated molecular patterns (PAMPs/MAMPs) and is subject to spatiotemporal regulation during development [169]. The D287 residue of the BAK1 protein is essential for this cleavage; the BAK1^D287A^ mutation completely blocks cleavage, leading to abnormal plasma membrane localization of BAK1, reduced phosphorylation capacity toward BIK1, and complete loss of function in core biological processes such as PTI, BR signaling, and cell death regulation [169]. Furthermore, *BAK1* overexpression-induced cell death depends on SOBIR1 but not on BIK1, and proteolytic cleavage of BAK1 alleviates this excessive immune response [48,169]. These findings demonstrate that proteolytic cleavage represents a novel and important layer in regulating the multi-functional homeostasis of BAK1. BIR3 is a kinase-inactive LRR-RK negative regulator in *Arabidopsis* that functions by directly binding to BAK1 and ligand-bound receptors such as BRI1/FLS2, competitively inhibiting BAK1 receptor complex formation while simultaneously stabilizing BAK1/BKK1 proteins [23]. Through this dual mechanism, BIR3 negatively regulates BR signaling, PTI, and cell death. The *bir3* single mutant exhibits weak phenotypes, whereas the *bak1 bir3* double mutant, due to destabilization of BAK1/BKK1, phenocopies the autoimmune death phenotype of *bak1 bkk1*, revealing that BIR3 serves as a core molecular switch balancing BAK1 signaling output and protein homeostasis [23]. MEMBRANE STEROID-BINDING PROTEIN1 (MSBP1) is a membrane steroid-binding protein localized to the plasma membrane and endocytic vesicles. It specifically binds to the extracellular domain of BAK1 in a BL-independent manner, promotes BAK1 endocytosis, and reduces the abundance of BAK1 at the plasma membrane, thereby inhibiting the formation of the BRI1-BAK1 complex and ultimately negatively regulating BR signaling [21]. These results indicate that BAK1 is likely degraded via the vacuolar trafficking pathway. The speculation is supported by the fact that KINASE-ASSOCIATED PROTEIN PHOSPHATASE (KAPP) promotes the entry of phosphorylated SERK1 (homologue of BAK1) into the endocytic pathway for degradation or recycling [170].

Previous studies have demonstrated that BAK1 activity and stability is modulated by multiple pathogen effectors, which in turn affect the dual biological processes of plant growth/development and immune responses. By directly targeting BAK1, the *P. syringae* effectors AvrPto and AvrPtoB block flg22-induced FLS2-BAK1 complex formation and inhibit BRI1-BAK1-mediated BR signaling, thereby broadly suppressing multiple MAMP-triggered immunity independently of FLS2 [98]. Further studies have revealed that AvrPtoB can simultaneously bind to both BAK1 and FLS2 through its N-terminal domain, and utilize its C-terminal E3 ubiquitin ligase activity to catalyze the polyubiquitination of the FLS2 intracellular domain, thereby mediating FLS2 degradation via the 26S proteasome pathway [97]. During this process, BAK1 is not degraded but is specifically bound by AvrPtoB, which inhibits its kinase activity (Table 1) [96]. Together, this dual mechanism blocks the BAK1-involved PTI immune response. HopB1 is a novel serine protease type III effector protein from *P. syringae*. It constitutively binds to FLS2 and, only after BAK1 is activated by flg22, specifically cleaves the activated form of BAK1, thereby blocking the PTI response. This process depends on BAK1 kinase activity and does not trigger host effector-triggered immunity (ETI) [51,166,167,171]. Unlike AvrPtoB and HopB1, PsTry1 is an apoplastic trypsin-like serine protease secreted by *Phytophthora sojae*. It is secreted into the host apoplast via a signal peptide, where it specifically binds to and cleaves the extracellular domain of BAK1, leading to the disassembly of the pattern recognition receptor (PRR)-BAK1 complex and thereby inhibiting PTI [172].

Recent studies showed that the stability of BAK1/SERKs is monitored by the plant nucleotide-binding leucine-rich repeat receptors (NLRs) surveillance system. The autoimmunity and cell death caused by loss of BAK1/BKK1 in *Arabidopsis* are mediated by the coiled-coil domain containing NLR (CC-NLR) ACTIVATED DISEASE RESISTANCE 1 (ADR1) [51]. The effector HopB1 cleaves activated BAK1/SERKs to mimic the autoimmunity and cell death phenotype, and this process is also dependent on ADR1, demonstrating that BAK1 and its homologs are “guardee targets” monitored by NLRs [51]. CONSTITUTIVE SHADE AVOIDANCE 1 (CSA1), a TOLL/INTERLEUKIN RECEPTOR (TIR)-type NLR receptor in *Arabidopsis*, physically binds BIR3 and monitors the homeostasis of the BAK1-BIR3 protein complex [166,167]. Upon disruption of the BAK1/BIR3 complex by effector proteins, homeostasis is perturbed, resulting in de-repression of CSA1 and subsequent activation of ETI-mediated cell death [166,167]. The newly identified LRR-RLK BTL2 acts as another key sentinel that monitors the integrity of BAK1/SERK4. Upon loss or disruption of BAK1/SERK4, BTL2 activates the CNGC20 calcium channel and subsequently triggers ADR1-dependent autoimmunity and cell death [51,173]. This pathway, along with the CSA1-mediated pathway, constitutes two parallel, independent but functionally complementary surveillance mechanisms monitoring BAK1 integrity.

While the E3 ubiquitin ligase directly responsible for regulating BAK1 stability has not yet been identified, multiple E3 ligases capable of directly interacting with BAK1 have been reported successively. The *Arabidopsis* U-box E3 ubiquitin ligases PUB12 and PUB13 constitutively interact with BAK1 [174,175]. Following flg22 treatment, BAK1 phosphorylates PUB12/PUB13, facilitating their recruitment to FLS2. PUB12/PUB13 then specifically ubiquitinate FLS2, leading to its degradation through the 26S proteasome pathway, thereby negatively regulating PTI immune responses [175]. The *Arabidopsis* PUB30 and PUB31 are U-box-type E3 ubiquitin ligases that negatively regulate the ERECTA signaling pathway [176]. Acting as a co-receptor and scaffold protein, BAK1 phosphorylates PUB30/31, thereby enhancing their interaction with ERECTA and mediating the ubiquitination and degradation of ERECTA. Furthermore, the protein stability of BAK1 itself is not affected by PUB30/PUB31 [176]. In the Nod factor receptor 5 (NFR5)-LjBAK1 module of *Lotus japonicus*, LjBAK1 interacts with LjPUB13/LjPUB7 and facilitates LjPUB13/LjPUB7-mediated ubiquitination of NFR5, thereby potentially regulating the protein stability and activity of NFR5 [66,177,178]. In *Nicotiana benthamiana*, NbBAK1 acts as a positive regulator of PTI by competing with NbSNIPER2a/b for binding to NbBIR2. This competition blocks the ubiquitination and proteasomal degradation of NbBIR2, leading to its stabilization and enhanced assembly of the NbFLS2-NbBAK1 immune complex [179]. While an E3 ubiquitin ligase responsible for BAK1 interaction and stability regulation remains to be discovered, mass spectrometry-based ubiquitinomic approaches have successfully mapped multiple ubiquitination sites on core BR signaling regulators, including BAK1 and SERK5 [180].

Considering the multifaceted roles of BAK1 in regulating plant growth and development, identification of E3 ubiquitin ligases that govern BAK1 stability is of great significance for deciphering the underlying signaling network. Although several E3 ligases have been reported to interact with BAK1, most cases describe BAK1-mediated modulation of the activity of these E3s. In contrast, only a small number of E3 components are known to directly modify BAK1 via ubiquitination to control its protein abundance. Further characterization of such direct regulators will substantially advance our mechanistic understanding of BAK1-centered signaling.

## 5. Ubiquitination in BIK1 Signaling

BIK1 is a receptor-like cytoplasmic kinase first identified in *Arabidopsis* as a regulator of resistance to *Botrytis cinerea* [181]. It acts as a central hub, linking diverse receptor complexes with downstream signaling components that regulate plant growth and development and cell death [100,182,183,184]. However, BIK1 is well known for acting downstream of many PRRs, such as FLS2, ELONGATION FACTOR-TU (EF-Tu) RECEPTOR (EFR), PEP1 RECEPTOR1 (PEPR1), CHITIN ELICITOR KINASE1 (CERK1), LYSIN-MOTIF RECEPTOR KINASE5 (LYK5), and BAK1, and for triggering a key signaling output of PTI [15,183,185,186,187]. In the absence of pathogen infection, BIK1 associates with plasma membrane-localized PRRs. When plants recognize MAMPs, the PRR complex assembles and mediates the phosphorylation of BIK1. The phosphorylated BIK1 then dissociates from the PRR complex and phosphorylates multiple proteins to regulate PTI signaling responses. These include membrane-localized proteins such as RBOHD [188,189], the calcium-permeable channel OSCA1.3 [190], the cyclic nucleotide-gated channels CNGC2/CNGC4 [191] and CNGC20 [192], Ca^2+^/H^+^ exchangers CAX1 and CAX3 [193], the extra-large G protein 2 (XLG2) [102], DIACYLGLYCEROL KINASE5 (DGK5) [194], as well as the nucleus-localized proteins WRKY33/50/57, RESISTANT TO PSEUDOMONAS SYRINGAE 4B (RPS4B) and RESISTANCE TO RALSTONIA SOLANACEARUM 1 (RRS1) [186,195].

**Figure 4:**
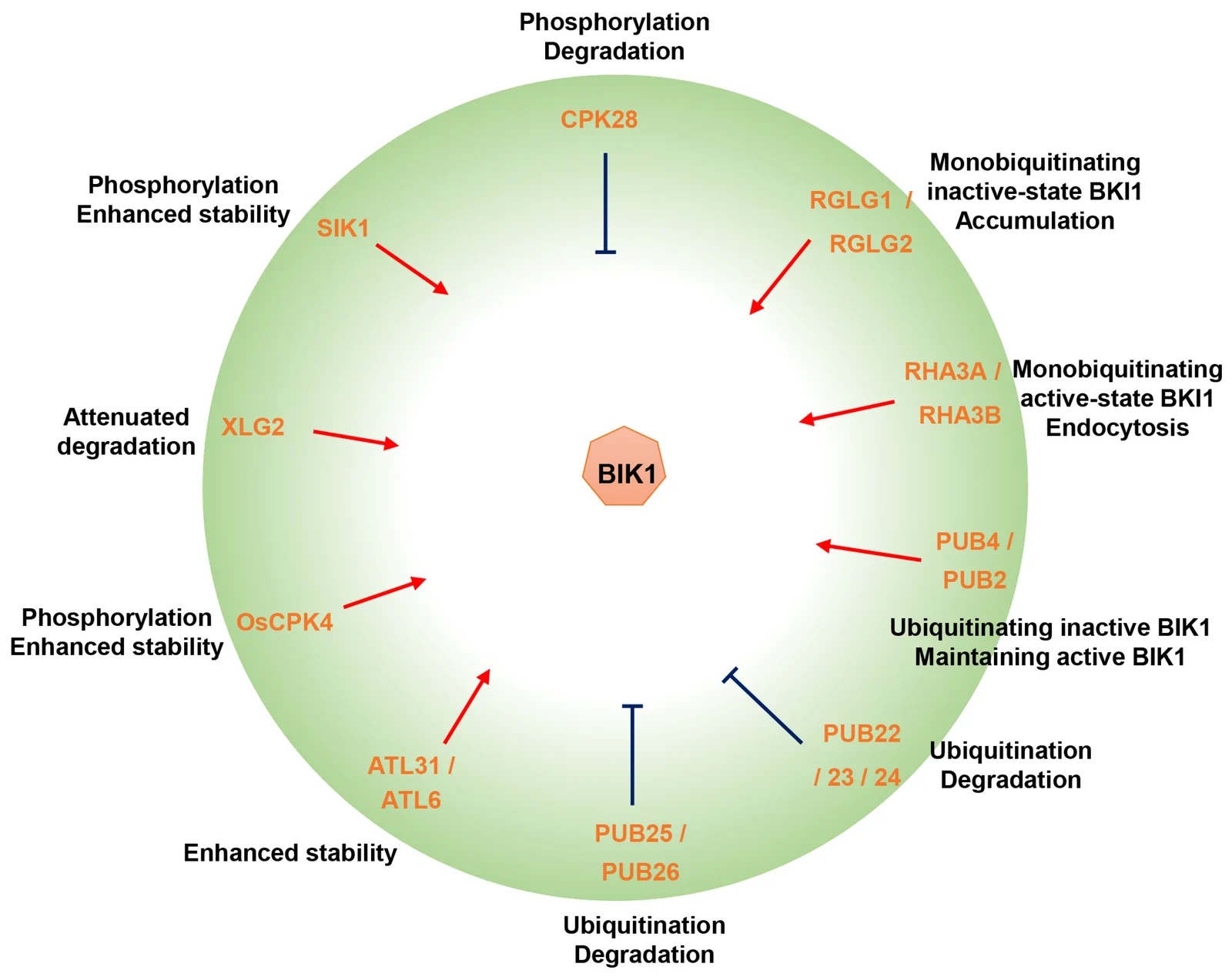
Regulation of BIK1 stability by ubiquitin-associated regulators. CPK28 phosphorylates BIK1 to induce BIK1 degradation, whereas SIK1 and OsCPK4 phosphorylate BIK1 to increase BIK1 stability. ATL31 and ATL6 ubiquitinate CPK28 for 26S proteasome-dependent degradation, thereby stabilizing BIK1. XLG2 interacts with inactive BIK1 and probably protects it from PUB25/26/22/23/24/4-mediated degradation in the immune rest state. Upon PAMP perception by PRRs, PUB4 cooperates with PUB2 to maintain the homeostasis of active BIK1; furthermore, RHA3A and RHA3B monoubiquitinate active BIK1 and promote its endocytosis, thereby activating immune signaling. Distinct from RHA3A and RHA3B, RGLG1 and RGLG2 monoubiquitinate inactive BIK1 and suppress PUB25-mediated degradation of BIK1, leading to the accumulation of BIK1. The T-shaped lines represent negative regulation of target protein stability and accumulation, whereas arrows represent positive regulation of target protein stability and accumulation.

Accordingly, the stability of BIK1 is precisely regulated to maintain immune signaling homeostasis (Figure 4 and Table 1). In *Arabidopsis*, CALCIUM-DEPENDENT PROTEIN KINASE 28 (CPK28) interacts with and phosphorylates BIK1, promoting its degradation via the 26S proteasome pathway, thereby buffering immune signaling intensity and maintaining immune homeostasis [100]. In contrast, MITOGEN-ACTIVATED PROTEIN KINASE KINASE KINASE KINASE (MAP4K) SERINE/THREONINE KINASE 1 (SIK1) interacts with the heterotrimeric G protein XLG2 to cooperatively attenuate the proteasome-mediated degradation of BIK1, thereby precisely regulating plant immune homeostasis [101,102]. Similarly, in rice, under non-pathogenic stress conditions, non-phosphorylated CALCIUM-DEPENDENT PROTEIN KINASE4 (OsCPK4) promotes the degradation of RECEPTOR-LIKE CYTOPLASMIC KINASE 176 (OsRLCK176), a close homolog of BIK1, to suppress excessive immune responses [103]. Upon pathogen infection, OsCPK4 and OsRLCK176 undergo mutual phosphorylation, which activates immune signaling and relieves the degradation of OsRLCK176, thereby initiating disease resistance responses [103]. Recent studies have shown that nitric oxide (NO) enhances the stability of the BIK1 protein through S-nitrosylation, inhibiting its degradation via the 26S proteasome pathway, thereby positively activating PTI [196]. BIK1 homeostasis is also regulated by the pathogen effector AvrPphB from *P. syringae*, which directly cleaves BIK1 via its cysteine protease activity, blocking downstream immune signaling and suppressing PTI responses [197].

To date, a number of E3 ubiquitin ligases have been confirmed to be involved in regulating the stability of the BIK1 protein. For instance, upon flg22 induction, plasma membrane-localized RING E3 ligases *ARABIDOPSIS* TO’XICOS EN LEVADURA31 (ATL31) and ATL6 ubiquitinate CPK28, promoting its 26S proteasome degradation and stabilizing BIK1 protein [104]. E3 ubiquitin ligases that interact with BIK1 and directly regulate its stability have also been reported. It has been demonstrated that the E3 ubiquitin ligases PUB25 and PUB26 specifically target the inactive, non-phosphorylated form of BIK1, promoting its degradation via the 26S proteasome pathway and thereby negatively regulating plant immunity and disease resistance [105]. The calcium-dependent kinase CPK28 phosphorylates PUB25/26 at the conserved sites T95/T94, enhancing their E3 ligase activity and accelerating BIK1 degradation [105]. In contrast, heterotrimeric G proteins (XLG2/XLG3, AGB1, and AGG1/AGG2) directly inhibit the E3 activity of PUB25/26 to stabilize BIK1 [105]. Together, these three components form a regulatory module that fine-tunes BIK1 protein abundance and the proportion of its activated form to maintain immune homeostasis in plants [105]. Moreover, the highly phosphorylated, active form of BIK1 evades PUB25/26-mediated degradation, ensuring effective immune signal transduction [105]. Further studies revealed that PUB22, PUB23, PUB24, PUB25, and PUB26 exhibit functional redundancy. The conserved threonine residues in all five proteins can be phosphorylated by CPK28 in vitro, and each of them specifically ubiquitinates inactive BIK1. In vitro cell-free degradation assays confirmed that these five PUB proteins collectively regulate BIK1 homeostasis and negatively regulate plant PTI through a redundant mechanism to prevent immune hyperactivation [105,106]. *Arabidopsis* PUB4 is another E3 ubiquitin ligase that specifically ubiquitinates and degrades inactive BIK1 [108]. Interestingly, upon immune activation, PUB4 cooperates with PUB2 to sustain the accumulation of active BIK1 [107]. Additionally, both proteins act together as scaffold factors to promote the FLS2-BIK1 and BIK1-RbohD interactions, thereby positively regulating PAMP-induced PTI responses [107]. The dissociation of phosphorylated BIK1 from the PRR complex is critical for initiating downstream immune responses [183], while this process is precisely regulated by the E3 ubiquitin ligases RING-H2 FINGER A3A (RHA3A) and RHA3B [109]. Upon recognition of flg22 by plants, BIK1 phosphorylation is initially triggered [109,183]; subsequently, the phosphorylated BIK1 undergoes specific monoubiquitination mediated by RHA3A/3B. This modification strictly depends on BIK1’s plasma membrane localization and its prior phosphorylation activation [109]. Notably, BIK1 monoubiquitination does not regulate its own protein stability but primarily controls its dissociation from the FLS2-BAK1 receptor complex and further mediates BIK1 endocytosis into endosomes, thereby activating downstream ROS burst and MAPK signaling pathways, ultimately enhancing plant resistance against pathogenic bacteria [109]. Furthermore, this monoubiquitination modification is conserved within the BIK1 family, as the homologous proteins PBL1 and PBL10 also undergo similar modifications. Mechanistically, BIK1 contains nine lysine residues that can be ubiquitinated. Simultaneously mutating these nine sites to generate the BIK1^9KR^ mutant completely abolishes BIK1 monoubiquitination, resulting in the loss of its physiological function in mediating plant immune responses [109]. In addition to RHA3A/3B, the E3 ubiquitin ligases RING DOMAIN LIGASE 1 (RGLG1) and RGLG2 also mediate monoubiquitination of BIK1 [110]. However, unlike RHA3A/3B, RGLG1/2 preferentially bind to the hypophosphorylated form of BIK1, directly mediating its monoubiquitination. This modification promotes the interaction between BIK1 and BAK1, suppresses PUB25-mediated ubiquitination and degradation of BIK1, thereby stabilizing BIK1 accumulation and positively regulating ROS burst and disease resistance immunity in plants [110]. Meanwhile, PUB25 ubiquitinates and degrades RGLG2, forming a mutually antagonistic regulatory loop [110]. The functions of RGLG1/2 depend on their own ubiquitin ligase activities and operate independently of the pathways involving RHA3A/3B-mediated BIK1 dissociation and PUB25-mediated BIK1 polyubiquitination and degradation, collectively fine-tuning the intensity of plant immune signaling [105,109,110]. Together, these findings suggest that the stability of BIK1 in plants is maintained by multiple regulatory mechanisms, corroborating previous observations that BIK1 contains numerous ubiquitination sites in vivo [180].

Dynamic ubiquitination of BIK1 is critical for immune homeostasis; however, the contextual transition between its degradative and non-degradative ubiquitination remains elusive. How BIK1 ubiquitination crosstalks with phosphorylation to balance immunity and growth also requires further exploration.

## 6. Ubiquitination in BSKs Signaling

BSKs constitute subfamily XII of receptor-like cytoplasmic kinases and function as core components in *Arabidopsis* BR signaling [25,198]. They modulate BR signal transduction via physical interactions with receptor BRI1 and downstream phosphatase BSU1 [25,198]. Twelve BSK paralogs (BSK1–BSK12) exist in *Arabidopsis*, whereas the rice genome encodes five homologs: OsBSK1-1, OsBSK1-2, OsBSK2 to OsBSK4 [199,200]. Members of the BSK family exhibit functional redundancy in BR signal regulation [199,200,201,202]. In addition to their role in modulating BR signaling, BSKs perform multifaceted functions in plant growth and development. For example, in *Arabidopsis*, BSK1/2 work with *Brassicaceae*-specific SSP to govern early embryonic polarity via the YODA MAPKK kinase pathway [203]. In rice, OsBSK2 forms homo/heterodimers with OsBSK3/4 to promote grain shape independently of canonical BR signaling [204]; together with OsBSK3, it directly interacts with OsMKKK10 and activates the MAPK cascade to positively control grain size and leaf angle [205]. BSK family proteins also function as positive regulators of plant innate immunity. BSK1 interacts with FLS2 to mediate flg22-triggered PTI and SA-mediated broad-spectrum disease resistance largely independent of canonical BR signaling [206,207], activates MAPK15/MAPKKK5 via phosphorylation for pathogen defense [207,208,209], and also receives immune phosphorylation from RLK902 through an FLS2-BAK1-BIK1- and flg22-PTI-independent pathway [210]. Furthermore, BSK family proteins are implicated in a wide range of abiotic stress responses. Under nitrogen-limiting conditions, *Arabidopsis* BSK3 promotes root elongation redundantly with BSK4/7/8, and their quadruple mutant displays severely impaired root and lateral root growth under low nitrogen [211]; BSK5 responds to salt, drought, and ABA, modulates auxin signaling and shade avoidance, and its mutant is hypersensitive to salt and ABA yet drought-tolerant [212,213]. Different from *Arabidopsis* BSK5, maize ZmBSK1 phosphorylates ZmCCaMK to activate antioxidant pathways and enhance drought resistance [214]. Overall, BSK paralogs undergo substantial functional divergence, coordinating BR signaling, immunity, and BR-environment crosstalk linked to nitrogen nutrition, light, salt, and drought, collectively serving as core kinases for plant external signal sensing and integration.

Considering the distinct physiological roles of BSK proteins, their stability is subject to rigorous regulation (Table 1). Recent studies have demonstrated that *Arabidopsis* BSK1 functions as a negative regulator of ABA signaling [99]. The protein homeostasis of BSK1 is antagonistically modulated by the E3 ubiquitin ligases PUB25/26 and the deubiquitinase UBP24. PUB25/26 promote BSK1 degradation via ubiquitination, while UBP24 sustains BSK1 stability through deubiquitination [99]. Exogenous ABA triggers the accumulation of PUB25/26, which competitively displaces UBP24 from BSK1. This breaks the dynamic equilibrium and accelerates BSK1 turnover, thereby alleviating the repression of ABA signaling imposed by BSK1 [99]. Meanwhile, UBP24 also mediates the deubiquitination of PUB25/26 to maintain their protein abundance, forming a dual regulatory loop that amplifies ABA signaling [99]. In addition, ABA induces BAK1-mediated phosphorylation and activation of PUB25/26, which elevates their E3 ligase activity and further accelerates BSK1 degradation [99]. Of note, PUB25 and PUB26 can mediate the ubiquitination of BAK1 in vitro, yet their functional reliability and physiological relevance have not been verified in planta [99]. Collectively, these interconnected pathways synergistically govern plant responses to ABA.

Existing research has successfully identified eight ubiquitination modification sites among five BSK family proteins [180]. Nevertheless, the biological consequences of most of these modification sites are still poorly resolved; it remains unclear how they respond to BR signals or environmental stimuli. In addition, numerous regulatory factors controlling BSK protein stability remain to be uncovered. A systematic characterization of these regulatory components and their target sites will help refine and fully delineate the upstream-downstream interactive regulatory network governing BSK-mediated signal transduction.

## 7. Ubiquitination in BIN2 Signaling

BIN2, a member of the glycogen synthase kinase 3 (GSK3) family, is a serine/threonine/tyrosine protein kinase and serves as a core negative regulator of the BR signaling pathway [215]. In addition to targeting the BR-downstream transcription factor BES1/BZR1, BIN2 can also phosphorylate numerous other substrate proteins, which in turn modulate the stability, subcellular distribution, and biological activity of these targets. Through these multifaceted regulatory functions, BIN2 coordinates multiple-hormone signaling cascades and a broad spectrum of biological processes, consequently shaping plant growth, development, and resistance responses against biotic and abiotic stresses [216,217,218,219,220]. The kinase activity and protein stability of GSK3-like proteins, including BIN2, are also modulated by a variety of interacting partners. For instance, in *Arabidopsis*, the histone deacetylase 6 (HDA6) physically interacts with BIN2, mediates deacetylation at the K189 residue of BIN2, and suppresses its kinase activity, consequently enhancing BR signaling [221]. ELONGATED HYPOCOTYL 5 (HY5) binds directly to BIN2 and augments its kinase activity, consequently driving the phosphorylation and subsequent degradation of BZR1 [222]. In rice, qGL3, a homolog of *Arabidopsis* BSU1, physically interacts with OsGSK3 (the rice homolog of BIN2) and catalyzes OsGSK3 dephosphorylation to sustain its protein stability; this functional pattern differs from that of BSU1 [25,223,224]. In wheat, physical interaction between CLAVATA1 (TaCLV1) and TaSG-D1 (the wheat homolog of BIN2) enables TaCLV1 to phosphorylate TaSG-D1, which ultimately results in the degradation of TaSG-D1 [225]. In addition to the aforementioned deacetylation and phosphorylation modifications, the kinase activity and protein abundance of BIN2 are modulated by the ubiquitination pathway [223], and multiple E3 ubiquitin ligases responsible for this regulatory process have been characterized.

The F-box protein KIB1 directly binds BIN2 and suppresses its activity by sterically blocking substrate binding and triggering BIN2 ubiquitination and proteasomal degradation (Table 1) [111,223]. In the BR pathway, BSU1 attenuates BIN2 kinase activity [25] and facilitates the KIB1-BIN2 interaction [111]. The BIN2 Y200F mutation does not interfere with their binding, while the dominant bin2-1 (E263K) mutation greatly impairs this interaction, accounting for the constitutive inhibitory activity of BIN2 in *bin2-1* mutants [25,111]. KIB1, KIB2, KIB3 and KIB4 are homologous genes with functional redundancy [111]. BRASSINOSTEROID F-BOX PROTEIN 1 (BRFP1) and BRFP2 are two additional F-box family proteins that interact with BIN2, confirmed by IP-MS and yeast two-hybrid analyses [112,113]. Previous studies have demonstrated that overexpression of *BRFP1* markedly reduces the protein abundance of BIN2 [113]. However, the detailed molecular mechanism underlying BRFP1/2-mediated ubiquitination and degradation of BIN2 remains to be further elucidated. Moreover, it is still unclear whether gain-of-function mutations of BIN2 can weaken its interaction with BRFP1 and BRFP2 [111,113,226,227]. OsGSK2, a rice homolog of BIN2, acts as a core negative regulator in the BR signaling pathway and can be degraded upon BR treatment [114,228,229]. Studies have revealed that BR induces the accumulation of the U-box E3 ubiquitin ligase TAIHU DWARF1 (TUD1) [114,230]. TUD1 directly interacts with OsGSK2 and mediates its ubiquitination and degradation, thereby activating the BR signaling pathway [114]. Recent studies have found that SALT TOLERANCE RECEPTOR-LIKE CYTOPLASMIC KINASE 1 (STRK1) interacts with and phosphorylates OsGSK2 at the Tyr223 residue [115,231]. This modification blocks the recognition and binding of OsTUD1 to OsGSK2, thereby maintaining the stability and accumulation of OsGSK2 [115]. The stabilized OsGSK2 subsequently continuously phosphorylates OsBZR1 and negatively regulates the BR signaling pathway [115,228,229]. Other studies have shown that the E3 ubiquitin ligase CONSTITUTIVE PHOTOMORPHOGENIC 1 (COP1) interacts with BIN2. It not only inhibits the kinase activity of BIN2, but also blocks BIN2-mediated phosphorylation and degradation of PIF3 via steric hindrance [116]. Notably, this regulatory process does not alter the protein stability of BIN2 [116]. A wealth of prior investigations has uncovered this special regulatory paradigm: E3 ubiquitin ligases tune the functional activity of substrates by modification, independent of any changes in substrate protein stability [232,233,234,235].

Ubiquitin-mediated proteasomal degradation of BIN2 is essential for BR signaling, yet the coordinated actions of E3 ligases and deubiquitinases controlling BIN2 stability remain incompletely defined. How ubiquitination modulates BIN2 activity and how ubiquitination integrates with BIN2 phosphorylation under diverse stimuli also merit further exploration.

## 8. Ubiquitination in BES1/BZR1 Signaling

BES1/BZR1 are plant-specific transcription factors that act as central components of the BR signaling pathway, governing the expression of BR-responsive target genes [28,29,37]. Beyond canonical BR signaling, BES1 and BZR1 function as key downstream regulators that mediate a wide range of endogenous cues and environmental stimuli [236,237]. It is known that BES1 and BZR1 undergo rapid interconversion between phosphorylated and dephosphorylated forms under the antagonistic regulation of BIN2 and PP2A; this process represses or promotes the transcriptional activity and protein stability of BES1/BZR1, thereby governing the output magnitude of BR signaling [35,215].

**Figure 5:**
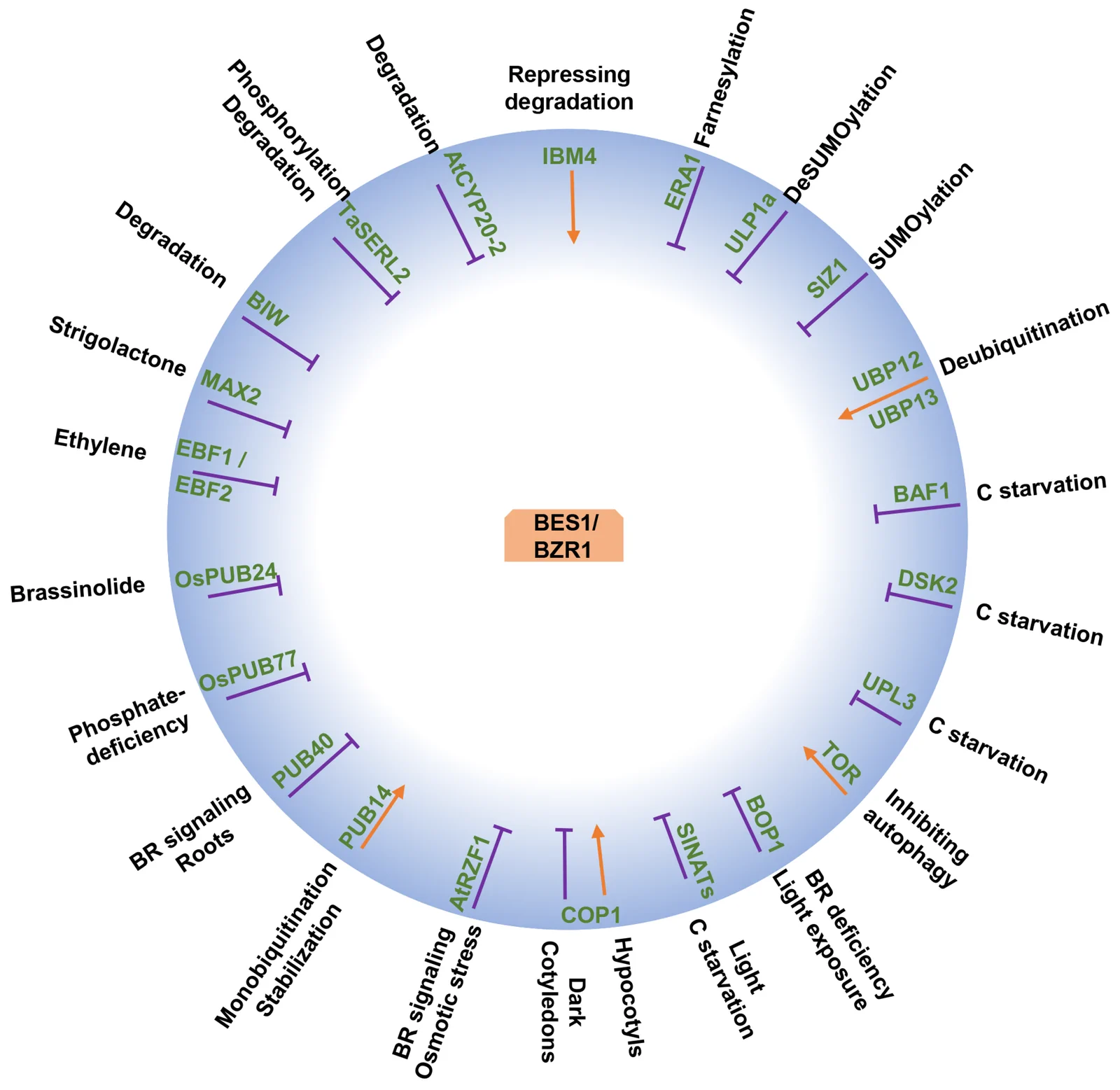
Regulation of the stability of BES1/BZR1 family proteins by ubiquitin-associated regulators. IBM4 promotes BR signaling by disrupting the interaction between BIN2 and BES1/BZR1, thereby inhibiting BIN2-dependent degradation of BES1 and BZR1. AtCYP20-2 interacts with BZR1 to facilitate its phosphorylation and degradation. TaSERL2 interacts with and phosphorylates TaBZR2 to accelerate its proteasome-dependent degradation. BIW inhibits BR signaling by interacting with BZR1 to mediate its degradation. SL activates MAX2 to ubiquitinate and degrade BES1 and BZR1, resulting in the inhibition of shoot branching. EBF1 and EBF2, two negative regulators of ethylene signaling, mediate the ubiquitination and subsequent degradation of BZR1, thereby repressing hook formation and cell elongation. In rice, OsPUB24 suppresses BR signaling by mediating the ubiquitination and subsequent degradation of OsBZR1, whereas in *Arabidopsis*, PUB40 specifically promotes the ubiquitination and degradation of BZR1 in roots. Phosphate deficiency triggers OsPUB77-dependent degradation of OsBZR3, repressing BR signaling and causing compact plant architecture. By contrast, BZR1 is monoubiquitinated and stabilized by PUB14, leading to an increased ovule number. AtRZF1 cooperates with BEH3 to negatively regulate BR signaling and osmotic stress. In darkness, COP1 potentiates BR signaling by targeting phosphorylated BZR1 for 26S proteasome-dependent degradation. Under shade or high-temperature conditions, however, COP1 confers organ-specific regulation of BES1 stability: it directly ubiquitinates BES1 to trigger its proteasomal degradation in cotyledons yet promotes BES1 accumulation in hypocotyls via an indirect mechanism. Under light or BR-deficiency conditions, induced BOP1 interacts with BZR1, inhibiting its nuclear import and thereby repressing BR signaling. Meanwhile, under light conditions, SINAT1/2/3/4 accumulate to negatively regulate BR signaling via the degradation of phosphorylated BES1. Under carbon-starvation conditions, BAF1 functions redundantly with SINAT1/2/3/4 and MAX2 to mediate the ubiquitination of BES1/BZR1 and trigger their autophagic degradation. Under carbon-sufficient conditions, TOR represses BZR1 autophagic degradation, and DSK2 represses BES1 autophagic degradation. By contrast, carbon starvation-triggered TOR inactivation permits UPL3-dependent autophagic degradation of BZR1. UBP12 and UBP13 physically interact with BES1/BZR1 and stabilize them via deubiquitination. SIZ1 physically interacts with and SUMOylates BES1, thereby accelerating BES1 degradation. By contrast, SUMO-modified BZR1 exhibits reduced degradation. DeSUMOylation of BZR1 mediated by ULP1a facilitates its degradation via a BIN2-dependent pathway. ERA1 facilitates BES1 degradation by enhancing its ubiquitination. The T-shaped lines represent negative regulation of target protein stability and accumulation, whereas arrows represent positive regulation of target protein stability and accumulation.

In addition to BIN2 and PP2A, numerous proteins have been proven to regulate the activity and protein stability of BES1/BZR1, thereby mediating plant growth, development, and stress responses (Figure 5 and Table 1). For instance, *Arabidopsis* IMB4 positively regulates BR signaling via direct binding to BES1/BZR1, which competitively interferes with BIN2-BES1/BZR1 interaction and alleviates BIN2-triggered ubiquitin-dependent degradation of BES1/BZR1 [36]. *Arabidopsis* CYCLOPHILIN 20-2 (AtCYP20-2) induces unfolding of the secondary structure of BZR1 and elevates its phosphorylation level, thereby facilitating the ubiquitin-mediated degradation of BZR1 [117]. Wheat somatic embryogenesis receptor-like kinase 2 (TaSERL2) mediates the phosphorylation of TaBZR2, a wheat homolog of *Arabidopsis* BES1/BZR1, which accelerates TaBZR2 turnover through the 26S proteasome system and compromises its transcriptional capacity [118]. In tomato, the BZR1-interacting WD40 protein (BIW) directly binds BZR1 and mediates its ubiquitin-mediated degradation, thereby negatively regulating BR signaling, lateral root formation, and chilling tolerance [119]. Recent studies in rice have revealed that high nitrate suppresses salt-induced OsGSK1-mediated phosphorylation of OsBZR3, thereby promoting the nuclear translocation of OsBZR3 from the cytoplasm [237]. Nuclear-localized OsBZR3 activates OsJAZ4 transcription, modulates root cell-wall remodeling and ion homeostasis, and enhances rice growth recovery under sustained salt stress, acting as a central hub integrating nitrate, BR, and JA signaling to regulate rice salt tolerance [237]. These studies also demonstrate that the ubiquitin–proteasome system is indispensable for precisely tuning the abundance of BES1 and BZR1, and the regulatory mechanisms governing the stability of BES1/BZR1 are diverse and sophisticated. Consistent with this notion, numerous E3 ubiquitin ligases that modulate the stability and multifaceted biological activities of BES1 and BZR1 have been characterized. These ligases act as pivotal regulators of tissue-specific growth programs and coordinate plant responses to a wide spectrum of hormonal and environmental signals.

MORE AXILLARY GROWTH 2 (MAX2) functions as an essential F-box component of the SCF ubiquitin E3 ligase complex and is a central regulator of strigolactone (SL) signaling in *Arabidopsis* [238]. Following SL perception by receptor DWARF14 (AtD14), MAX2 mediates polyubiquitination and 26S-proteasome-dependent degradation of both phosphorylated and dephosphorylated BES1/BZR1 [120]. As a transcriptional activator promoting shoot branching, reduced BES1 abundance represses branching-related BRANCHED1 (BRC1) and SL biosynthetic genes *MAX3*/*MAX4*, thus inhibiting axillary bud outgrowth [120]. The F-box proteins EIN3 BINDING F-BOX PROTEIN1 (EBF1) and EBF2 are known negative regulators of ethylene signaling [121,239,240]. Recent work reveals their new role in BR signaling [121]. EBF1 interacts with BZR1 and triggers its 26S proteasome-dependent degradation, which suppresses BZR1 target genes for cell elongation and weakens BR-driven hypocotyl growth and apical hook formation [121].

Four recent studies demonstrate that the stability of BES1 family proteins is controlled by four U-BOX-type E3 ubiquitin ligases, namely OsPUB24, OsPUB77, PUB40 and PUB14. Each of these ligases separately regulates leaf inclination, plant architecture, root development, and reproductive growth in response to different environmental cues [122,123,124,125]. OsPUB24, a rice U-BOX E3 ligase, represses BR signaling by mediating OsBZR1 degradation via the 26S proteasome [122]. OsSK22-dependent phosphorylation stabilizes OsPUB24, whereas BR represses OsSK22 to trigger OsPUB24 self-degradation and OsBZR1 accumulation, and the *ospub24* mutant with enlarged leaf angles and BR hypersensitivity verifies that this module governs rice plant architecture [122]. Under phosphate-deficient conditions, *RLI1a* expression is suppressed, relieving its repression of *OsPUB77* and causing OsPUB77 accumulation [123]. OsPUB77 then ubiquitinates OsBZR3 for degradation by the 26S proteasome, thereby inhibiting BR signaling [123,237]. This results in reduced leaf angles and fewer tillers, facilitating rice adaptation to low phosphate [123]. In *Arabidopsis*, PUB40 mediates the root-specific degradation of BZR1 [124]. PUB40 preferentially binds to phosphorylated BZR1 and promotes its ubiquitination [124]. The *bzr1-1D* mutation weakens this interaction, allowing BZR1 accumulation across all root cell layers. BIN2 phosphorylates PUB40, enhancing its stability and substrate affinity, whereas BR signaling suppresses BIN2 and promotes PUB40 degradation [124]. Genetic evidence demonstrated that the *pub39 pub40 pub41* triple mutant exhibits low-phosphate tolerance due to BZR1 accumulation [124]. Unlike OsPUB24, OsPUB77, and PUB40, which mediate the ubiquitination and degradation of BES1 family proteins and thus function as negative regulators [122,123,124], PUB14 acts as a positive key factor in the BR pathway that regulates ovule development and seed yield [125]. PUB14 directly binds to and monoubiquitinates BZR1, thereby maintaining BZR1 protein stability and activating the ovule developmental program [125]. In addition, physical association of PUB14 with BZR1 competitively inhibits the PUB40-BZR1 interaction, representing an alternative mechanism by which PUB14 sustains BZR1 protein abundance [124,125]. Genetic evidence shows that *PUB14* overexpression promotes BZR1 accumulation and elevates ovule number, while *PUB14* loss-of-function accelerates BZR1 degradation, represses ovule initiation, and thereby decreases seed yield [125]. Evolutionary analyses reveal that the PUB14-BZR1 regulatory module is conserved in multiple plant species, including *Brassica napus*, *Petunia hybrida*, and tomato, which offers new insights for high-yield and stress-resistant crop molecular breeding [125]. Analogously, another E3 ubiquitin ligase, *Arabidopsis thaliana* RING ZINC FINGER 1 (AtRZF1), mediates BR-dependent stress responses in *Arabidopsis*; AtRZF1 encodes a C3H2C3-type E3 ubiquitin ligase, and loss-of-function *atrzf1* mutants accumulate elevated proline and exhibit enhanced drought tolerance [126,241]. As a BES1/BZR1-family transcription factor, *BES1*/*BZR1 HOMOLOG 3* (*BEH3*) loss further boosts the osmotic stress resistance of *atrzf1*, while *BEH3* overexpression increases plant stress sensitivity [126,241]. Although these genetic phenotypes are well-established, direct biochemical proof that AtRZF1 regulates BEH3 protein stability is missing, and the core mechanism of the AtRZF1–BEH3 module remains unclear.

Light is an essential prerequisite for plant growth and development. As core regulatory components governing plant growth and development, BES1/BZR1 proteins are modulated by several light-responsive E3 ubiquitin ligases, which mediate light regulation of BR signaling via controlling the stability of BES1/BZR1 proteins [128,129,242]. COP1 is a RING-type E3 ubiquitin ligase [116,128,232,243]. Under darkness, activated COP1 targets phosphorylated BZR1 for degradation, elevating the abundance of dephosphorylated BZR1 [128]. This facilitates the formation of active BZR1 homodimers or BZR1-PHYTOCHROME INTERACTING FACTOR 4 (PIF4) heterodimers, thereby activating BR signaling and promoting hypocotyl elongation during skotomorphogenesis [128]. In the light, *phytochrome* B (*phyB*) loss-of-function mutations mimic the dark response by reducing the accumulation of phosphorylated BZR1 and consequently triggering BR signal activation [128]. *Arabidopsis* contains five members of the RING-type E3 ubiquitin ligase SEVEN IN ABSENTIA OF *ARABIDOPSIS THALIANA* SINAT (SINAT) family. Among them, SINAT1, SINAT2, SINAT3, and SINAT4 possess ubiquitin ligase activity, whereas SINAT5 is catalytically inactive due to the absence of the RING domain [244,245]. Light suppresses the autoubiquitination and subsequent degradation of SINAT1/2/3/4, thereby elevating their protein abundance. Accumulated SINATs reduce the pool of active dephosphorylated BES1/BZR1 under light and repress BR signaling, ultimately restraining hypocotyl elongation [129]. This regulatory mechanism complements the dark-specific pathway in which COP1 mediates the degradation of phosphorylated BZR1 [128]. Together, these two pathways precisely govern the switch between photomorphogenesis and skotomorphogenesis by differentially controlling the stability of BES1/BZR1 variants with distinct phosphorylation statuses. Under shade or high temperature conditions, activated COP1 physically interacts with BES1 and facilitates its degradation via the 26S proteasome in cotyledons [127]. By contrast, in hypocotyls, COP1 elevates BES1 abundance by degrading unidentified repressors of BES1 [127]. This finding unveils the tissue-specific dual functionality of COP1 and underscores the sophisticated ubiquitin-mediated regulatory network governing BES1/BZR1 proteins during plant adaptation to environmental fluctuations. BLADE-ON-PETIOLE 1 (BOP1), also termed BRZ-SENSITIVE-SHORT HYPOCOTYL1 (BSS1), is a bric-a-brac/tramtrack/broad (BTB) domain-containing protein and a core component of CUL3-type E3 ubiquitin ligases [131,246]. Under BR deficiency or light exposure, *BOP1* expression is induced, and the protein assembles into cytoplasmic oligomeric complexes [131]. These cytoplasmic oligomers directly interact with BZR1, sequestering the transcription factor to prevent its nuclear import, thereby lowering the nuclear pool of active dephosphorylated BZR1 and ultimately repressing the transcription of BR-responsive genes [131]. However, since BOP proteins function as substrate adaptors for CUL3-based E3 ubiquitin ligases [247], the specific BOP1-containing E3 complexes that modulate the turnover of BES1/BZR1, along with their intrinsic molecular regulatory mechanisms, still await systematic elucidation.

Ubiquitin-modified proteins have two degradation fates: one is degradation via the 26S proteasome pathway; the other is the initiation of ubiquitin-dependent selective autophagy, whereby autophagy sorting receptors recognize ubiquitin tags, encapsulate target substrates into autophagosomes, and deliver them to lysosomes for degradation [248,249]. In addition to degradation via the 26S proteasome pathway, BES1/BZR1 can also be degraded through autophagy [130,132,133,134]. Plants sense sugar availability via the TARGET OF RAPAMYCIN (TOR) kinase to modulate the stability of BZR1, a master transcription factor governing growth [132,250]. Under sufficient sugar supply, activated TOR represses autophagy, thereby blocking BZR1 degradation; accumulated BZR1 synergizes with BR signaling to drive cell elongation [132]. Conversely, sugar starvation or TOR inactivation triggers autophagy-dependent degradation of BZR1, leading to growth arrest [132]. Recent studies have revealed that the HECT-type E3 ubiquitin ligase UBIQUITIN PROTEIN LIGASE 3 (UPL3) is an essential factor for the degradation of BZR1 under sugar starvation [133,251]. Upon sugar starvation or TOR kinase inactivation, UPL3 protein accumulates abundantly and mediates the ubiquitin-dependent degradation of BZR1 [133]. The reduction in BZR1 abundance blocks BR-driven growth programs, lowering carbon consumption to sustain plant survival under starvation stress [132,133]. In the absence of UPL3, BZR1 continuously accumulates and constitutively activates growth, rapidly depleting carbon reserves and ultimately causing widespread seedling mortality [133]. In light of the documented functions of HECT-family E3 ubiquitin ligases in autophagy [252], it is hypothesized that UPL3 regulates the degradation of BZR1 via the autophagy route, necessitating more in-depth mechanistic research. Analogously, BES1 participates in plant responses to both drought stress [61,253] and carbon starvation stress [130]. Upon exposure to drought or carbon starvation, the ubiquitin receptor protein DOMINANT SUPPRESSOR OF KAR2 (DSK2) forms complexes with BES1 and the autophagy-related protein AUTOPHAGY-RELATED PROTEIN 8 (ATG8) simultaneously, which shuttles BES1 to undergo autophagic degradation [130]. Stress-induced activation of BIN2 drives the phosphorylation of DSK2, which reinforces DSK2–ATG8 interaction and expedites the autophagic removal of BES1 [130]. Conversely, *DSK2* loss-of-function mutants exhibit robust BES1 buildup under adverse conditions. Sustained BR growth signaling consequently overconsumes carbon and water reserves, leading to a striking decline in seedling survival during drought and carbon limitation [130]. Furthermore, upon treatment with the autophagy inhibitor E64d [130,254], comparative analysis of BES1 abundance between wild-type and *SINAT RNAi* materials reveals attenuated ubiquitination of BES1 in *SINAT*-deficient plants during starvation [130]. This evidence supports the conclusion that SINAT family E3 ubiquitin ligases are indispensable for driving the ubiquitin-dependent selective autophagic turnover of BES1 under carbon starvation stress. The F-box E3 ubiquitin ligase BES1-ASSOCIATED F-BOX1 (BAF1) constitutes another protein that drives autophagy-mediated degradation of BES1 under carbon starvation [134]. Carbon starvation triggers robust transcriptional induction of *BAF1*; the resulting BAF1 protein directly interacts with BES1 and catalyzes its K63- and K48-linked polyubiquitination. BAF1 preferentially targets BES1 for degradation via DSK2-dependent selective autophagy, thereby repressing BR-governed plant growth [134]. Loss of *BAF1* function leads to sustained accumulation of BES1 protein. Under prolonged sucrose starvation, mutant plants excessively consume endogenous carbon reserves and exhibit markedly reduced survival rates. In contrast, *BAF1* overexpression confers enhanced plant tolerance to carbon limitation [134]. In addition to modulating BES1 protein homeostasis, BAF1 also regulates the stability of its homologous transcription factor BZR1, yet it acts through a distinct regulatory mechanism compared with the HECT-type E3 ligase UPL3 [133,134]. Under sucrose starvation or TOR-inhibited conditions, the *baf1* mutant accumulates elevated levels of phosphorylated BZR1, whereas the *upl3* mutant exhibits increased abundance of dephosphorylated BZR1 [133]. Consistent with these biochemical distinctions, hypocotyl elongation of *baf1* seedlings is comparable to wild-type controls or slightly suppressed, and markedly shorter than that of *upl3* single mutants [133]. As expected, the hypocotyl length of *baf1 upl3* double mutants falls in an intermediate range between the two single mutant lines [133]. Of note, under light or dark conditions, the *baf1 max2* and *baf1 SINAT RNAi* double mutants exhibit longer hypocotyls than their respective single mutants (*max2* and *SINAT RNAi*), indicating functional redundancy between BAF1 and MAX2, as well as between BAF1 and SINAT family E3 ligases [120,129,133,134].

Recent studies have further uncovered additional enzymes governing BES1/BZR1 protein homeostasis. Among them, the deubiquitinating enzymes UBIQUITIN-SPECIFIC PROTEASE 12 (UBP12) and UBP13 are capable of interacting with both phosphorylated and dephosphorylated BES1/BZR1 isoforms [136,255]. By cleaving K48- and K63-linked ubiquitin chains, UBP12 and UBP13 block the entry of BES1 into the 26S proteasome and selective autophagy pathway, sustaining the abundance of cytoplasmic phosphorylated BES1 and nuclear active BES1 [135,136]. Intriguingly, the deubiquitinating enzymes UBP12 and UBP13 can interact with UPL3 to form a functional complex, which coordinately modulates the protein turnover of core carbon metabolism substrates [256]. The SAF-A/B, Acinus, and PIAS (SAP) and Msx-interacting-zinc finger (MIZ) DOMAIN-CONTAINING LIGASE1 (SIZ1), a SUMO E3 ligase, directly interacts with BES1 and mediates its sumoylation at the K302 residue [137,257]. This modification accelerates BES1 degradation via the 26S proteasome pathway and impairs its DNA-binding and transcriptional activation capacities simultaneously [137]. Contrary to BES1, SUMOylation of BZR1 blocks its physical interaction with BIN2, thereby alleviating BZR1 phosphorylation and degradation, facilitating BZR1 nuclear translocation, and boosting its transcriptional activity to sustain normal plant growth [138]. Meanwhile, salt stress induces substantial accumulation of SUMO protease UBIQUITIN-LIKE-SPECIFIC PROTEASE 1A (ULP1a), which mediates the deSUMOylation of BZR1 at K280 and K310 [138]. DeSUMOylated BZR1 is readily recognized and degraded by BIN2, consequently attenuating BR signaling and triggering growth suppression to facilitate plant salt resistance [138]. Whether ULP1a exerts regulatory effects on BES1 remains to be further investigated. The farnesyltransferase ENHANCED RESPONSE TO ABA 1 (ERA1) acts as a negative regulator of the BR signaling pathway [139]. It indirectly elevates the ubiquitination level of BES1 to facilitate its degradation via the 26S proteasome, thereby suppressing BR signal output [139]. Loss of ERA1 function leads to massive accumulation of BES1 and renders plants hypersensitive to BRs [139].

These studies reveal that the ubiquitin-mediated degradation pathway of BES1/BZR1 exerts crucial and sophisticated regulatory functions during plant environmental adaptation. Numerous ubiquitination sites of BES1/BZR1 are still unidentified, and their responses to diverse environmental conditions as well as biological functions remain to be dissected; the molecular basis underlying non-proteolytic ubiquitination on BES1/BZR1 also deserves attention, calling for comprehensive multi-dimensional mechanistic analysis.

## 9. Concluding Remarks and Future Perspectives

Since the identification of the plasma-membrane BR receptor BRI1 and co-receptor BAK1 over two decades ago [7,17,18,30], plant BR signaling has been extensively elucidated with numerous core signaling factors characterized [4,258,259,260]. Accumulating evidence shows that these central modulators function critically in canonical BR pathways as well as various other signaling routes through BR-dependent or BR-independent regulatory mechanisms. Ubiquitination-mediated regulation of the stability of core BR signaling components represents a vital molecular mechanism governing plant growth, development, immunity, and stress responses upon endogenous developmental cues and exogenous environmental stimuli. As multifunctional regulators, components in BR signaling are subjected to targeted ubiquitination, which tunes their stability, endocytosis, subcellular localization, and molecular activity to confer unique regulatory capabilities. Recent studies have demonstrated that E3 ubiquitin ligases not only govern the turnover and functionality of their substrate proteins but are also subject to reciprocal regulation of their own stability and activity by substrates, for example via phosphorylation mediated by protein kinases. This mutual regulatory interplay underpins a sophisticated mechanism for fine-tuning signaling outputs, enabling plants to mount adaptive responses under diverse growth environments (Table 1). As an increasing number of E3 ubiquitin ligases that modulate the stability of BR signaling components have been identified, substantial progress has been achieved in research on the regulation of BR signaling component stability via ubiquitination modification; nevertheless, numerous scientific questions remain unresolved.

First, numerous E3 ubiquitin ligases targeting core BR signaling components, including BRI1, BIN2, BIK1, and BES1/BZR1, have been identified in previous studies. Nevertheless, the molecular mechanisms underlying the substrate recognition motifs and interacting domains remain largely uncharacterized. Moreover, research on deubiquitination-mediated reverse regulation of ubiquitinated substrates is limited. The dynamic balance between ubiquitination and deubiquitination, as well as its regulatory logic under various stress conditions, requires further systematic investigation.

Second, the current literature lacks sufficient documentation regarding E3 ligase- and DUB-mediated regulation of BAK1, BSKs, BSU1 and BKI1. Identification of novel E3 ubiquitin ligases will fill these unresolved gaps and enable the assembly of an integral ubiquitin regulatory atlas of the BR signaling pathway.

Third, it is necessary to dissect competitive recognition of individual BR proteins by multiple E3 ligases and their stage- and stress-specific functions. Distinct ubiquitination sites modified by different E3s target substrates to the 26S proteasome, selective autophagy, or vacuolar degradation. Combined tissue and stress expression analyses will uncover the redundancy and synergy of these E3 ligases in roots, stems, and leaves under drought, saline-, alkali-, and temperature-related stresses, establishing a spatiotemporal regulatory landscape of BR ubiquitination.

Fourth, existing research demonstrates that homologous E3 ubiquitin ligase genes display pronounced functional divergence, and even opposing functions, across crop species, exemplified by wheat ZnF, rice TT3.1, and foxtail millet SiSGD1. These homologous proteins integrate environmental and developmental signals to trigger target protein ubiquitination, yet their core evolutionary regulatory mechanisms are poorly understood. Comprehensive research on this topic will unravel their molecular regulatory networks and decipher the molecular basis of crop-specific environmental adaptability.

Fifth, beyond ubiquitination, other post-translational modifications such as phosphorylation, SUMOylation, and farnesylation also affect the stability of substrate proteins. However, how these modifications determine the preferential recognition of substrates such as BES1/BZR1 by distinct E3 ligases, and how they coordinate with ubiquitination to regulate protein stability and activity, remains to be further elucidated.

Finally, systematic research on specific E2-E3 pairs in crops is lacking, impeding the precise prediction of substrate recognition, ubiquitin chain topology, and regulatory effects. It is essential to characterize how E3s selectively recruit E2s in response to fluctuating environmental and developmental signals. Future integration of high-resolution structural analysis, comparative evolutionary genomics, and in vivo biochemical assays will reveal co-evolutionary constraints and functional plasticity of E2-E3 pairs, uncover molecular mechanisms of crop species-specific adaptation, and provide a foundation for clarifying hierarchical regulation of ubiquitination cascades governing BR signaling pathways.

## Figures and Tables

**Table 1:** Ubiquitin-associated regulators of core BR-signaling components.

Targets	Regulators	Protein Type	Effect on Targets	Reference
BRI1	TASH3	Subunit of TPLATE adaptor complex	Mediates endocytosis of ubiquitinated BRI1 for degradation	[82]
	BIL4	TGN/EE localized protein	Interacts with BRI1 in endosomes and prevents its vacuolar degradation to enhance BR signaling	[83]
	OsELT1	Kinase-dead receptor-like kinase	Interacts with OsBRI1 and suppresses its polyubiquitination and degradation to enhance BR signaling	[84]
	SYP22	Subunit of SNARE complex	Interacts with BRI1 and recycles internalized BRI1 to enhance BR signaling	[85]
	VAMP727			
	LAZ1	tonoplast-localized DUF300 proteins	Blocks vacuolar trafficking and turnover of BRI1 to enhance BR signaling	[86]
	LAZ1H1			
	PUB12	U-box E3 ligase	Mediates BRI1 polyubiquitination and subsequent degradation to attenuate BR signaling	[87]
	PUB13	U-box E3 ligase		
	UBC35	UBC E2	Mediates the K63 ubiquitination of BRI1 and subsequent degradation	[75]
	UBC36	UBC E2		
	UBP12	Deubiquitinating enzyme	Deubiquitinates K63-linked polyubiquitination of BRI1 and inhibits its turnover to enhance BR signaling	[88]
	UBP13	Deubiquitinating enzyme		
	Desi3a	Desumoylating isopeptidase	Mediates BRI1 deSUMOylation and subsequent degradation to attenuate BR signaling	[89]
	EMR	RING E3 ligase	Mediates the degradation of bri1-5 and bri1-9 to attenuate BR signaling	[90]
	UBC32	UBC E2		
	PAWH1	Subunit of HRD1 complex	mediates the degradation of bri1-5 and bri1-9 to attenuate BR signaling	[91]
	PAWH2			
	OsSMG3	UBC E2	Polyubiquitinates misfold OsBRI1 to trigger its degradation and to enhance BR signaling	[92]
	OsDGS1	RING E3 ligase		
	SiUBC32	UBC E2	Polyubiquitinates BRI1 to enhance its stability in a nonproteolytic manner and to enhance BR signaling	[93]
	SiSGD1	RING E3 ligase		
BKI1	PUB30	U-box E3 ligase	Polyubiquitinates BKI1 to trigger its degradation	[94]
	TaZnF	RING E3 ligase		[95]
BAK1	AvrPtoB	E3 ligase	Inhibits BAK1 kinase activity in a nonproteolytic manner	[96,97,98]
	PUB25	U-box E3 ligase	Mediates the polyubiquitination of BAK1 in vitro	[99]
	PUB26	U-box E3 ligase		
BIK1	CPK28	Calcium-dependent protein kinase	Phosphorylates BIK1 to trigger its degradation via the 26S proteasome	[100]
	SIK1	MAP4K	Interacts with and attenuates the proteasome-mediated degradation of BIK1	[101]
	XLG2	G protein		[101,102]
	OsCPK4	Calcium-dependent protein kinase	Phosphorylates OsRLCK176 (a close homolog of BIK1) to enhance its stability	[103]
	ATL31	RING E3 ligase	Triggers CPK28 breakdown and inhibits ubiquitin-dependent BIK1 degradation	[104]
	ATL6	RING E3 ligase		
	PUB25	U-box E3 ligase	Polyubiquitinates inactive BIK1 to trigger its degradation	[105]
	PUB26	U-box E3 ligase		
	PUB22	U-box E3 ligase		[106]
	PUB23	U-box E3 ligase		
	PUB24	U-box E3 ligase		
	PUB4	U-box E3 ligase	Polyubiquitinates inactive BIK1 for degradation and protects active BIK1 against breakdown	[107,108]
	PUB2	U-box E3 ligase		[107]
	RHA3A	RING E3 ligase	Monobiquitinates active-state BIK1 to trigger its endocytosis	[109]
	RHA3B	RING E3 ligase		
	RGLG1	RING E3 ligase	Monobiquitinates inactive-state BIK1 to trigger its accumulation in a nonproteolytic manner	[110]
	RGLG2	RING E3 ligase		
BSK1	PUB25	U-box E3 ligase	Polyubiquitinates BSK1 to trigger its degradation	[99]
	PUB26	U-box E3 ligase		
	UBP24	Deubiquitinating enzyme	Deubiquitinates BSK1 and promotes its accumulation	
BIN2	KIB1	F-box protein	Blocks BIN2-substrate binding and triggers BIN2 polyubiquitination	[111]
	BRFP1	F-box protein	Interacts with BIN2 and triggers its protein degradation	[112,113]
	BRFP2	F-box protein		
	OsTUD1	U-box E3 ligase	Mediates OsGSK2 (a close homolog of BIN2) polyubiquitination and subsequent degradation	[114]
	OsSTRK1	Receptor-like cytoplasmic kinase	Disrupts OsTUD1–OsGSK2 interaction and enhances OsGSK2 protein stability	[115]
	COP1	RING E3 ligase	Inhibits BIN2 kinase activity in a nonproteolytic manner	[116]
BES1/BZR1	IMB4	Importin-β family protein	Interacts with BES1/BZR1 to block BIN2 association and repress its ubiquitin-dependent degradation	[36]
	AtCYP20-2	Cyclophilin protein	Interacts with BZR1 to promote ubiquitin-mediated degradation of BZR1	[117]
	TaSERL2	LRR-RLK	Phosphorylates TaBZR2 to accelerate its degradation via the 26S proteasome	[118]
	BIW	WD40-repeat protein	Binds BZR1 to facilitate its ubiquitin-dependent degradation	[119]
	MAX2	F-box protein	Mediates BES1/BZR1 ubiquitination and subsequent degradation	[120]
	EBF1	F-box protein	Interacts with BZR1 and triggers its proteasome-mediated degradation	[121]
	OsPUB24	U-box E3 ligase	Ubiquitinates OsBZR1 to trigger its degradation via the 26S proteasome	[122]
	OsPUB77	U-box E3 ligase	Mediates OsBZR3 ubiquitination and subsequent degradation	[123]
	PUB40	U-box E3 ligase	Polyubiquitinates BZR1 to trigger its degradation by the 26S proteasome in roots.	[124]
	PUB14	U-box E3 ligase	Monoubiquitinates BZR1 and inhibits the PUB40-BZR1 interaction to stabilize BZR1	[125]
	AtRZF1	RING E3 ligase	functions together with BEH3 (a homolog of BES1/BZR1) to modulate osmotic-stress and BR signaling	[126]
	COP1	RING E3 ligase	Interacts with phosphorylated BZR1 and triggers its degradation in darkness; interacts with BES1, triggering its degradation in the cotyledons and its accumulation in the hypocotyls under shade or warm temperature conditions	[127,128]
	SINAT1	RING E3 ligase	Polyubiquitinates dephosphorylated BES1 to trigger its degradation in the light; Polyubiquitinates BES1 for autophagic degradation under carbon-starvation conditions	[129,130]
	SINAT2	RING E3 ligase		
	SINAT3	RING E3 ligase		
	SINAT4	RING E3 ligase		
	BOP1	BTB domain containing protein	Interacts with BZR1 and inhibits its nuclear translocation	[131]
	TOR	Protein kinase	Inhibits the autophagic degradation of BZR1	[132]
	UPL3	HECT E3 ligase	Interacts with BZR1 and facilitates its degradation under sugar-starvation conditions	[133]
	DSK2	Ubiquitin receptor protein	Associates with BES1 and targets it for autophagic degradation upon drought and carbon-starvation conditions	[130]
	BAF1	F-box E3 ligase	Binds to BES1/BZR1 and marks it for degradation through the autophagy pathway when plants undergo carbon starvation	[134]
	UBP12	Deubiquitinating enzyme	Deubiquitinates BES1/BZR1 and promotes its stability	[135,136]
	UBP13	Deubiquitinating enzyme		
	SIZ1	SUMO E3 ligase	Interacts with BES1 and inhibits its protein stability and transcriptional activity by SUMOylation	[137]
	ULP1a	SUMO protease	DeSUMOylates BZR1 and facilitates its degradation mediated by BIN2	[138]
	ERA1	farnesyltransferase	Promotes the ubiquitination of BES1 and accelerates its degradation by the 26S proteasome	[139]

## Data Availability

No new data were created or analyzed in this study. Data sharing is not applicable to this article.
